# Neurobehavioral effects of fungicides in zebrafish: a systematic review and meta-analysis

**DOI:** 10.1038/s41598-023-45350-6

**Published:** 2023-10-24

**Authors:** Carlos G. Reis, Leonardo M. Bastos, Rafael Chitolina, Matheus Gallas-Lopes, Querusche K. Zanona, Sofia Z. Becker, Ana P. Herrmann, Angelo Piato

**Affiliations:** 1 Programa de Pós-Graduação em Neurociências, Instituto de Ciências Básicas da Saúde, Universidade Federal do Rio Grande do Sul (UFRGS), Porto Alegre, RS Brazil; 2 Laboratório de Psicofarmacologia e Comportamento (LAPCOM), Departamento de Farmacologia, Instituto de Ciências Básicas da Saúde, Universidade Federal do Rio Grande do Sul (UFRGS), Porto Alegre, RS Brazil; 3 Programa de Pós-Graduação em Farmacologia e Terapêutica, Instituto de Ciências Básicas da Saúde, Universidade Federal do Rio Grande do Sul (UFRGS), Porto Alegre, RS Brazil; 4 Laboratório de Neurobiologia e Psicofarmacologia Experimental (PsychoLab), Departamento de Farmacologia, Instituto de Ciências Básicas da Saúde, Universidade Federal do Rio Grande do Sul (UFRGS), Porto Alegre, RS Brazil; 5 Brazilian Reproducibility Initiative in Preclinical Systematic Review and Meta-Analysis (BRISA) Collaboration, Rio de Janeiro, Brazil; 6 Laboratório de Neurofisiologia e Neuroquímica da Excitabilidade Neuronal e Plasticidade Sináptica, Departamento de Bioquímica, Instituto de Ciências Básicas da Saúde, Universidade Federal do Rio Grande do Sul (UFRGS), Porto Alegre, RS Brazil

**Keywords:** Environmental impact, Ecology

## Abstract

Pesticides are widely used in global agriculture to achieve high productivity levels. Among them, fungicides are specifically designed to inhibit fungal growth in crops and seeds. However, their application often results in environmental contamination, as these chemicals can persistently be detected in surface waters. This poses a potential threat to non-target organisms, including humans, that inhabit the affected ecosystems. In toxicologic research, the zebrafish (*Danio rerio*) is the most commonly used fish species to assess the potential effects of fungicide exposure, and numerous and sometimes conflicting findings have been reported. To address this, we conducted a systematic review and meta-analysis focusing on the neurobehavioral effects of fungicides in zebrafish. Our search encompassed three databases (PubMed, Scopus, and Web of Science), and the screening process followed predefined inclusion/exclusion criteria. We extracted qualitative and quantitative data, as well as assessed reporting quality, from 60 included studies. Meta-analyses were performed for the outcomes of distance traveled in larvae and adults and spontaneous movements in embryos. The results revealed a significant overall effect of fungicide exposure on distance, with a lower distance traveled in the exposed versus control group. No significant effect was observed for spontaneous movements. The overall heterogeneity was high for distance and moderate for spontaneous movements. The poor reporting practices in the field hindered a critical evaluation of the studies. Nevertheless, a sensitivity analysis did not identify any studies skewing the meta-analyses. This review underscores the necessity for better-designed and reported experiments in this field.

## Introduction

Chemical pesticides are synthetical active ingredients used to control pests that may threaten the productivity of crops^1^. To yield high productivity levels, modern agriculture employs large amounts of pesticides^2^. In 2020, the global consumption of these products reached almost 3 million tonnes^3^. The substantial quantity and the method by which they are applied results in environmental contamination of the soil, surface waters, and food^4–6^. Data shows that less than 0.1% of the pesticide hits the intended target species, leaving the remaining residual impacting the environment and public health^7^. Its presence in superficial waters generates risk to the non-target organisms by decreasing biodiversity and the population of primary food chain producers and reducing the prey for the aquatic organisms^8–10^. Moreover, the dissemination of pesticides in the environment represents a risk to humans, whereas their presence in the water supply leads to potential consumption^11–14^.

According to the target organism, these substances can be classified as herbicides, insecticides, rodenticides, and fungicides^15^, being the fungicides one of the most used chemicals^16^. Their application aims to kill and/or inhibit fungal growth in agriculture, both in seeds and crops^17^.

Due to the need to understand the effects of exposure to these products, the scientific literature presents several studies with animals in this area^18^. The model organism zebrafish (*Danio rerio,* Hamilton 1822) is widely used in toxicology, mostly because of its high fecundity, fast development, transparency of the embryo, and high homology of organs and genetics concerning humans^19–21^. In addition, the zebrafish is an aquatic animal that dwells in potentially contaminated ecosystems, representing the eventual consequences of exposure to other cohabitant species^22^. It has been reported that exposure to fungicides in zebrafish causes behavioral, neurochemical, developmental, metabolic, hormonal, hepatotoxic, cardiotoxic, enzymatic, morphological, and molecular alterations^23–28^.

From 2012 to 2019, more than 100 articles were published investigating the effects of fungicides in zebrafish, which represents the second most investigated type of pesticide in this organism^29^. However, there is a high methodological heterogeneity between the studies. The interventions, developmental stages, and outcomes addressed are extremely variable between studies. Regarding the intervention, plenty of compounds used as fungicides exhibit distinct mechanisms of action^30^ and can be administered over a wide range of durations through multiple routes of administration. As for the developmental stage, in vivo exposure can be performed in embryos, larvae, or adults; the outcomes are distinctly selected according to the research question and the capabilities of the research group (neurotoxicity, hepatotoxicity, cardiotoxicity, among others)^31^.

Many studies were published on the toxic effects of fungicides on neurobehavioral parameters in zebrafish^22,32^. However, no secondary studies systematically synthesize these results to obtain an understanding supported by published evidence to optimize the planning of new research. An accurate description of these preclinical data and a meta-analysis can help avoid redundant studies and the consequent use of animals. Furthermore, considering the reproducibility issues raised for the zebrafish research field^33,34^, it is essential to identify possible sources of bias and conflicting results, including assessing the quality of available publications. This systematic review and meta-analysis of literature aimed at synthesizing the data from neurobehavioral effects of fungicide exposure in zebrafish, also analyzing reporting quality and publication bias.

## Methods

Before screening studies and data extraction, a protocol guiding this review was registered in Open Science Framework, and preregistration is available at https://osf.io/f2d38^35^. The reporting of this study follows the Preferred Reporting Items for Systematic Reviews and Meta-Analyses (PRISMA) guidelines^36^.

### Search strategy

The studies were identified through a search in the literature using three different databases: PubMed, Scopus, and Web of Science. The search strategies were designed to adapt to each database characteristics. The terms were combined for the intervention (fungicide exposure) and the population of interest (zebrafish), aiming to conduct a comprehensive search, including all the available articles that fulfilled the inclusion criteria. The complete query for each database can be found at https://osf.io/5ae9q^35^. The strategy did not apply any search filter, language restriction, or limit of year. The search was performed on the 1st of December, 2021, and the articles were imported to Rayyan software^37^ to identify and remove the duplicates.

### Study screening

Initially, the retrieved studies from the three databases were analyzed to filter and exclude duplicates (performed by CGR). The remaining articles were pre-selected based on their title and abstract. If a reason to exclude the record was not found, at this stage, it was carried forward to the full-text screening stage. In both stages (title/abstract and full-text), two independent reviewers (CGR and LMB, RC or SZB) examined each study. Disagreements between the decisions of the reviewers were resolved by a third reviewer (QKZ, AP, or APH).

Experimental studies evaluating the effects of exposure to fungicides in zebrafish on the following parameters were included: motor function, sensory function, learning and memory, social behavior, sexual behavior, eating behavior, anxiety-like or fear-related behaviors, behaviors related to the reward system, and behaviors related to circadian rhythms. The parameters were included only if they were linked to the central nervous system. The identity of the compound as a fungicide was consulted in the Pesticide Properties Database^38^.

In the first phase (screening of title/abstract), papers were excluded according to the following criteria:

1. Type of study design: reviews, comments, abstracts published in conference proceedings, corrections, editorials;
2. Type of population: in vitro investigations or studies with species other than zebrafish;
3. Type of intervention: biological and commercial formulations or mixtures of fungicides, non-interventional studies;

In the next phase (full-text screening), the following criteria were added, and the articles were excluded based on the above items plus:

4. Comparison: when there is no proper control group (same organism, same procedure, except for fungicide exposure);
5. Outcome measures: if there is no assessment of any previously cited neurobehavioral outcome.

More information about this section is available at https://osf.io/wmsvg.

### Data extraction

Two independent investigators (CGR and LMB, RC or SZB) performed the data extraction, and a discussion between the two reviewers resolved disagreements. The information and values of interest were directly extracted from the text and tables. When not possible, WebPlotDigitizer software (v4.5, Rohatgi, A., Pacifica, CA, USA, https://automeris.io/WebPlotDigitizer) was used to determine the values from the graphs manually. The following data were extracted: (1) study identification: study title, digital object identifier (DOI), first author, last author, year of publication, and last author affiliation; (2) model animal specifications: strain, sex, the developmental stage during exposure, age during exposure, the developmental stage during the test, age during the test; (3) fungicide exposure characteristics: fungicide, administration route and type (i.e., static, semi-static or flow through), frequency of renewal, frequency of exposure, duration of exposure, dose/concentration and the interval between exposure and test; (4) test properties: test nomenclature, category of measured variable (e.g., anxiety, locomotor, social) and the measured variable.

Regarding the authors of the studies, co-authorship networks were elaborated using VOSviewer software version 1.6.18 (https://www.vosviewer.com)^39,40^.

Data were collected for each variable according to the outcomes of interest, including the mean and the number of animals (n) for both the control and exposed groups. The standard deviation (SD) or standard error of the mean (SEM) was extracted for the reported mean value. If the SEM was reported, the SD was calculated by multiplying SEM by the square root of the sample size (SD = SEM ∗ √n).

In instances where the sample size was reported as a range, the lowest value was used. Whenever information was unclear or missing, attempts were made to contact the corresponding author of the study via email, with two separate attempts made at least two weeks apart.

### Reporting quality

To assess the reporting quality of included studies, two independent reviewers (CGR and LMB, RC, or SZB) evaluated each paper based on^41^, which proposes criteria for transparent reporting. The observed topics were: (1) mention of any randomization process; (2) sample size estimation; (3) mention of inclusion/exclusion criteria; (4) mention of any process to ensure blinding during the experiments. A score of “yes” or “no” was given for each topic, meaning that it was or was not reported, respectively. The outcome measurements performed by any automated software were considered blinded. Reporting quality plots were created using robvis^42^.

A complete guide for assessing the reporting quality associated with each item in this review is available at https://osf.io/uy5v3.

### Meta-analysis

To perform a meta-analysis, at least 5 studies with the same outcome were required a priori^35^. Whenever two or more experimental groups shared the same control, the sample size of the control group was divided by the number of comparisons and then rounded down. Further information about the basic aspects of our method can be found at^43^.

Effects sizes were determined with the standardized mean difference (SMD) using Hedge’s G method^44^. SMD was used because studies examined a common outcome while employing different measurement approaches, which makes it necessary to standardize the findings in a uniform scale to allow combination across studies. Briefly, SMD expresses the size of an intervention effect relative to the observed variability^45,46^. Analyses were conducted using R Project for Statistical Computing with packages meta^47^ (https://cran.r-project.org/package=meta) and ggplot2^48^ following Hedge’s random effects model, given the anticipated heterogeneity between studies. Values for SMD were reported with 95% confidence intervals. Heterogeneity between studies was estimated using I^2^^49^, τ^2^, and Cochran’s Q^50^ tests. Heterogeneity variance (τ^2^) was estimated using the restricted maximum likelihood estimator^51,52^. The confidence intervals around pooled effects were corrected using Knapp-Hartung adjustments^53^. Values of 25%, 50%, and 75% were considered as representing low, moderate, and high heterogeneity, respectively, for I^2^, and a p-value ≤ 0.1 was considered significant for Cochran’s Q. Prediction intervals were estimated and represent the range of effects expected for future studies^45^. Furthermore, a subgroup meta-analysis was performed to evaluate if the developmental stage of the animals was a potential source of heterogeneity. Studies were grouped into two categories: larval and adult. Subgroup analysis was only performed when there were at least five unique studies for each subgroup. A p-value ≤ 0.1 was considered significant for subgroup differences^54^.

We conducted an exploratory meta-analysis to investigate an association between the effect and the fungicide class by categorizing them based on their chemical structure. Even without reaching the minimum of 5 studies, we ran a meta-analysis with 4 articles investigating fungicides of the triazole and anilide groups.

A mixed-effects meta-regression analysis was conducted to explore the relationship between the effect sizes and fungicide concentration as a moderator variable. The random effects structure accounted for potential heterogeneity across studies^55^. Meta-regressions excluding studies based on the sensitivity analysis were also performed.

Publication bias was investigated by generating funnel plots and performing Duval and Tweedie’s trim and fill analysis^56^ and Egger’s regression test^57^. Analyses were only conducted when at least five studies were available within a given outcome for funnel plots and at least ten studies for the regression test. A p-value < 0.1 was considered significant for the regression test.

### Sensitivity analysis

A sensitivity analysis was conducted to assess if any experimental or methodological difference between studies was biasing the main effect found in the meta-analysis. Analyses were performed following the “leave-one-out jackknife method”^58^. A minimum of three comparisons were required for each outcome to conduct a sensitivity analysis. Furthermore, we conducted complementary meta-analyzes excluding studies that, when omitted in the leave-one-out, had observations that changed the overall effect direction. We also ran meta-analyses excluding studies containing experiments with atypically high SMD, as seen in the forest plots^59^.

## Results

### Search results

**Figure 1:**
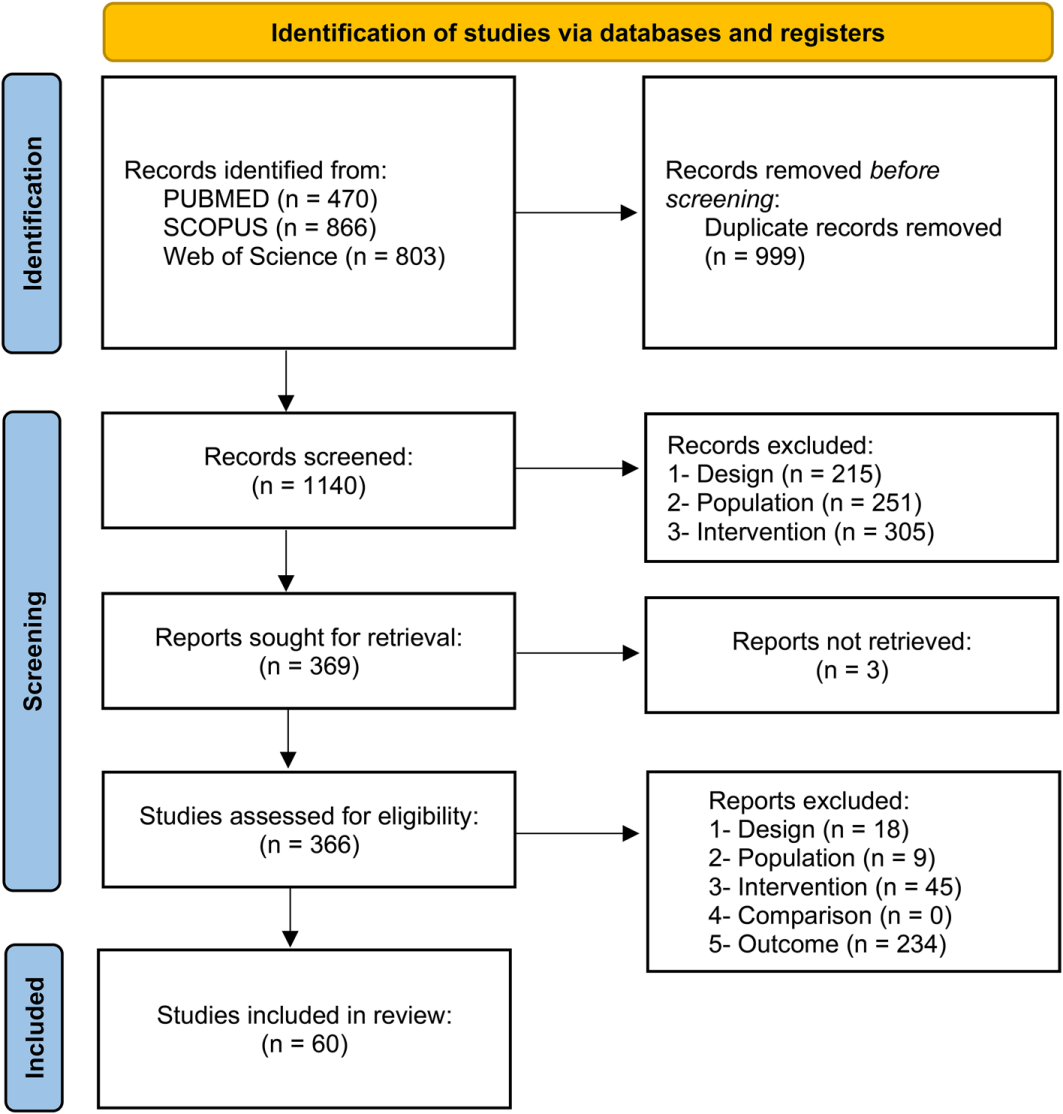
Flowchart diagram of the collection of studies and selection process.

The search in the three databases retrieved a total of 2139 results. After removing duplicates, 1140 articles were screened for eligibility by analyzing the titles and abstracts. As a result of the first screening phase, 369 studies remained to be assessed based on their full-text. At this phase, 3 were not retrieved, and 60 fulfilled the criteria and were included in the review (Fig. 1). The main overall reasons for the exclusions were outcome (n = 234), population (n = 260), and intervention (n = 350). Concerning the quantitative synthesis, 8 studies were excluded because the minimum number of studies to perform a meta-analysis was not reached for the reported outcomes and 10 because of missing information. There were 18 experiments measuring distance using luminous transitions (dark/light) in larvae that were not included due to the variations between the protocols^60^, which makes the comparison infeasible. This resulted in 24 studies included in the quantitative synthesis. Detailed reasons for excluding studies from the meta-analysis are available at https://osf.io/qpcew.

### Study characteristics

A qualitative description of the studies is provided in Table 1. The identification of the studies was attributed according to the table available at https://osf.io/85d2p. A total of 43 different fungicides were addressed in the articles included in this review. Studies with the fungicides difenoconazole (n = 5, 8.3%), boscalid (n = 4, 6.6%), and pyraclostrobin (n = 4, 6.6%) were the most frequent.

**Table 1:** Qualitative description of studies reporting effects of fungicide exposure on neurobehavioral and neurochemical outcomes in zebrafish. The indicated concentrations of exposure were used to assess the behavioral outcomes.

Study ID	Fungicide	Concentration (mg/L)	Duration of exposure	Developmental stage during exposure/outcome assessment	Main findings
Domingues et al.^82^	Prochloraz	0.3, 0.6, 1.2, 2.4, 4.8, 7.2, 9.6	96 h	Embryo/Larvae & Adult/Adult	**Locomotor behavior** ↑ Spontaneous movements ↓ Distance traveled ↑ Distance in the dark ↑ Distance in the light ↑ Velocity ↑ Acceleration ↑ Absolut turn angle **Neurochemical outcomes** ↓ ChE activity (larvae) ↑ GST activity (larvae)
Fitzmaurice et al.^83^	Benomyl	0.29	120 h	Embryo/Larvae	**Locomotor behavior** ↓ Distance traveled
Mu et al.^84^	Difenoconazole	0.5, 1,1.5, 2, 2.5, 3	24 h	Embryo/Embryo	**Locomotor behavior** ↑ Spontaneous movements (1.5 mg/L) ↓ Spontaneous movements (2.5, 3 mg/L) ↑ Reversal rate behavior
Andrade et al.^85^	Carbendazim	1.1, 1.19, 1.3, 1.41, 1.53, 1.66, 1.8	120 h	Embryo/Larvae	**Locomotor behavior** ↓ Distance in the light ↓ % Small distance in the light ↓ % Small distance in the dark ↑ % Long distance in the light ↑ % Long distance in the dark ↑ Swimming time in the light ↑ Swimming time in the dark **Neurochemical outcomes** ↑ ChE activity ↑ GST activity = CAT activity
Jin et al.^86^	Imazalil	0.01, 0.03, 0.1, 0.3	96 h	Embryo/Larvae	**Locomotor behavior** ↓ Distance traveled ↓ Distance in the dark ↓ Distance in the light ↓ Velocity**Neurochemical outcomes**↓ AChE levels ↓ AChE activity = DA levels
Lulla et al.^87^	Ziram	0.0003—0.305	7 days	Embryo/Larvae	**Locomotor behavior** ↓ Distance in the dark = Distance in the light ↓ Velocity
Mu et al.^88^	Difenoconazole	0.5, 2	96 h	Embryo/Embryo	**Locomotor behavior** = Spontaneous movements
Yang et al.^68^	Thifluzamide	2.66, 2.76, 2.85, 2.95, 3.04, 3.23 & 2.66, 2.76, 2.85, 2.95, 3.04 & 2.66, 2.85, 3.04, 3.23, 3.42, 3.61	96 h & 144 h & 96 h	Embryo/Embryo & Embryo/Larvae & Larvae/Larvae	**Locomotor behavior** ↓ Spontaneous movements (embryo) ↓ Swimming rate (larvae)
Yang et al.^89^	Flutolanil	1.5, 1.8, 2.16, 2.59, 3.1	24 h	Embryo/Embryo	**Locomotor behavior** ↑ Spontaneous movements
Altenhofen et al.^90^	Tebuconazole	1, 2, 4 & 1, 4, 6	120 h & 96 h	Embryo/Larvae & Adult/Adult	**Locomotor behavior** ↓ Distance traveled ↓ Absolut turn angle (larvae) = Crossings **Anxiety/fear-related behavior** ↓ Time in the periphery = Time in the upper zone **Aggressive behavior** ↑ Time in the bottom**Neurochemical outcomes**↓ AChE activity
De la Paz et al.^91^	Triadimefon	16	8 h	Larvae/Larvae	**Locomotor behavior** ↑ Locomotor activity
Costa-Silva et al.^92^	Mancozeb	1	23 h & 43 h	Embryo/Embryo & Embryo/Embryo dechorionated	**Locomotor behavior** ↑ Spontaneous movements ↑ Number of stimuli (embryo dechorionated) ↑ Response to touch (embryo dechorionated) **Neurochemical outcomes** = GST activity ↓ GSH levels = GPx activity
Fan et al.^93^	Hymexazol	417, 480, 554, 639, 738	48 h	Embryo/Larvae	**Locomotor behavior** ↓ Swimming rate
Li et al.^94^	Pyraoxystrobin	2.03, 2.44, 2.9, 3.51,4.22, 5.08	24 h	Embryo/Embryo	**Locomotor behavior** = Spontaneous movements
Qian et al.^95^	Boscalid	0.7, 2, 2.3, 2.6, 2.9, 3.2	22 h	Embryo/Embryo	**Locomotor behavior** ↑ Spontaneous movements
Teng et al.^96^	Difenoconazole	0.0005, 0.005, 0.05, 0.5	24 h	Embryo/Embryo	**Locomotor behavior** ↑ Spontaneous movements
Teng et al.^97^	Difenoconazole	0.0005, 0.005, 0.05, 0.5	24 h	Embryo/Embryo	**Locomotor behavior** ↑ Spontaneous movements
Wang et al.^98^	Fluazinam	0.04, 0.09, 0.13	6 days	Embryo/Larvae	**Locomotor behavior** ↑ Swimming activity in the dark (0.04 mg/L) ↓ Swimming activity in the dark (0.09, 0.13 mg/L) = Swimming activity in the light
Cao et al.^65^	Ziram	0.0003, 0.003	7 days	Embryo/Larvae	**Locomotor behavior** ↑ Swimming activity ↑ Distance traveled (0.003 mg/L) = Distance in the dark = Distance in the light = Total velocity ↑ Velocity in light **Anxiety/fear-related behavior** ↓ Time in the dark = Frequency in the dark
Cao et al.^99^	Cyproconazole	2.9, 7.2, 14.5, 29.1, 72.9, 145.8	24 h & 48 h & 7 days	Embryo/Embryo & Embryo/Larvae	**Locomotor behavior** ↓ Spontaneous movements ↓ Swimming activity in the dark = Swimming activity in the light
Cao et al.^100^	Maneb	0.02, 0.13, 0.26	7 days	Embryo/Larvae	**Locomotor behavior** ↓ Swimming activity in the dark ↓ Swimming activity in the light
Li et al.^67^	Pyraclostrobin	0.009, 0.018, 0.36	4 days	Larvae/Larvae	**Locomotor behavior** ↓ Distance traveled ↓ Velocity**Neurochemical outcomes**↑ Glutamate receptor activity
Paredes-Zúñiga et al.^63^	Triadimefon	5, 20, 35	10 h & 11 min	Larvae/Larvae & Adult/Adult	**Locomotor behavior** ↓ Swimming activity ↓ Distance traveled (larvae) ↑ Distance traveled (adult) ↑ Velocity **Anxiety/fear-related behavior** ↓ Time in the periphery ↑ Time in the bottom zone ↓ Time in the upper zone **Aggressive behavior** ↑ Number of bites**Neurochemical outcomes**↑ DA levels ↓ 5-HT levels
Perez-Rodriguez et al.^101^	Tebuconazole	0.03, 0.3, 3	6 days	Embryo/Larvae	**Locomotor behavior** ↓ Distance in the dark = Distance in the light = Velocity **Anxiety/fear-related behavior** ↑ Mean time in the dark = Cumulative time in the dark ↑ Frequency in the dark zone
Qian et al.^102^	Penthiopyrad	2.3, 2.4, 2.5, 2.6, 2.7, 2.8, 2.9 & 0.3, 0.6, 1.2	1 day & 5–8 days	Embryo/Embryo & Embryo/Larvae	**Locomotor behavior** ↑ Spontaneous movements (2.5, 2.6, 2.7 mg/L) ↓ Spontaneous movements (2.9 mg/L) ↓ Swimming activity ↓ Distance traveled ↓ Velocity ↓ Acceleration
Souders et al.^103^	Propiconazole	0.03, 0.3, 3.4	144 h	Embryo/Larvae	**Locomotor behavior** ↓ Distance traveled ↓ Distance in the dark
Teng et al.^104^	Propiconazole	0.5, 2.5, 4.5	24 h & 120 h	Embryo/Embryo & Embryo/Larvae	**Locomotor behavior** ↑ Spontaneous movements ↓ Distance traveled ↓ Velocity ↓ Swimming activity ↓ Acceleration
Tian et al.^105^	Prothioconazole	0.0375, 0.075, 0.15	24 h	Embryo/Embryo	**Locomotor behavior** = Spontaneous movements **Neurochemical outcomes** ↓ GSH levels = SOD activity = CAT activity ↑ MDA levels
Valadas et al.^106^	Propiconazole	0.000425, 0.00085, 0.0017, 0.0085	96 h	Adult/Adult	**Locomotor behavior** = Distance traveled ↓ Crossings **Anxiety/fear-related behavior** ↓ Time in the upper zone ↑ Time in the upper zone ↓ Entries in the upper zone = Entries in the bottom zone **Neurochemical outcomes** ↑ SOD activity ↑ CAT activity = MDA levels = SH levels = NPSH levels
Wang et al.^107^	Oxine-copper	0.01, 0.02, 0.04	24 h	Embryo/Embryo & Larvae/Larvae	**Locomotor behavior** ↓ Number of tail coiling ↓ Distance traveled ↓ Swimming activity ↓ Velocity ↑ Absolut turn angle**Neurochemical outcomes**↓ AChE activity(embryo) ↑ SOD activity (embryo) ↑ CAT activity (embryo) ↑ MDA levels (embryo) ↑ ROS levels (embryo)
Yang^108^	Flutolanil	0.125, 0.5, 2	24 h & 96 h	Embryo/Embryo & Embryo/Larvae	**Locomotor behavior** ↓ Spontaneous movements = Distance traveled **Neurochemical outcomes** ↑ DA levels
Yang^109^	Thifluzamide	0.19, 1.9, 2.85	24 h & 96 h	Embryo/Embryo & Embryo/Larvae	**Locomotor behavior** = Spontaneous movements = Distance traveled **Neurochemical outcomes** ↓ DA levels
Zhou et al.^110^	Captan	0.58, 0.66, 0.75, 0.86, 1.00, 1.16	24 h	Embryo/Embryo	**Locomotor behavior** = Spontaneous movements
Hussain et al.^111^	Tebuconazole & Dimethomorph & Difenoconazole	0.3 & 0.3 & 0.4	24 h	Larvae/Larvae	**Locomotor behavior** ↑ Distance in the dark (tebuconazole, dimethomorph) ↓ Distance in the dark (difenoconazole) ↑ Distance in the light ↑ Burst movement count in the light ↑ Burst movement count in the dark (dimethomorph, difenoconazole) ↑ Rotation count in the light (dimethomorph, difenoconazole) ↑ Rotation count in the dark (dimethomorph, difenoconazole)
Jia et al.^23^	Penconazole ( +) & Penconazole (-)	1, 2	24 h & 96 h	Embryo/Embryo & Embryo/Larvae	**Locomotor behavior** ↑ Spontaneous movements (( +)-penconazole) ↓ Velocity (( +)-penconazole) **Neurochemical outcomes** ↓ AChE activity (( +)-penconazole) ↓ DA levels (( +)-penconazole) ↓ 5-HT levels (( +)-penconazole) = Glycine levels = Norepinephrine levels
Kumar et al.^112^	Azoxystrobin & Pyraclostrobin	0.00001, 0.0001, 0.01, 0.1, 1	5 days	Embryo/Larvae	**Locomotor behavior** ↓ Distance traveled **Neurochemical outcomes** = MDA levels
Liu et al.^113^	Propamocarb	0.01, 0.1, 1	7 days	Embryo/Larvae	**Locomotor behavior** ↑ Distance traveled ↑ Distance in the dark = Distance in the light ↑ Velocity **Neurochemical outcomes** ↓ AChE activity = MDA levels ↓ DA levels = SOD activity ↑ CAT activity ↑ GPx activity ↓ GST activity
Pang et al.^114^	Myclobutanil	4, 6, 8, 10, 12, 14, 16	24 h	Embryo/Embryo	**Locomotor behavior** ↑ Spontaneous movements (4, 6, 8, 10, 12 mg/L) ↓ Spontaneous movements (16 mg/L)
Shen et al.^26^	Mepanipyrim	0.0001, 0.001, 0.01, 0.1	7 days	Embryo/Larvae	**Locomotor behavior** ↑ Distance traveled (7, 14 dpf) ↓ Distance traveled (14 dpf) ↑ Velocity (7, 14 dpf) ↓ Velocity (14 dpf) ↑ Acceleration = Absolut turn angle ↓ Immobile time (7 dpf) **Neurochemical outcomes** = AChE activity ↑ GABA levels
Souders et al.^115^	Triticonazole	0.3, 3.1, 31.7	6 days	Embryo/Larvae	**Locomotor behavior** ↑ Distance in the dark = Distance in the light
Tang et al.^66^	Cyprodinil	0.0001, 0.001, 0.01, 0.1	24 h	Embryo/Embryo	**Locomotor behavior** ↓ Spontaneous movements
Teng et al.^27^	Flutolanil	0.00025, 0.05, 1	60 days	Adult/Embryo (offspring)	**Locomotor behavior** = Spontaneous movements
Vasamsetti^116^	Etridiazole	3.75, 7.5, 15, 30, 60	96 h	Embryo/Larvae	**Locomotor behavior** ↑ Immobile time
Wang et al.^28^	Boscalid	5, 15, 25	24 h	Embryo/Embryo & Larvae/Larvae	**Locomotor behavior** ↓ Number of tail coiling ↓ Distance traveled ↓ Distance in the dark ↓ Distance in the light ↓ Velocity ↑ Absolut turn angle ↑ Immobile time**Neurochemical outcomes** = AChE activity ↑ MDA levels ↓ SOD activity ↑ CAT activity ↑ ROS levels
Zhang et al.^117^	Zoxamide	0.16, 0.33, 0.84, 1.68	24 h & 6 days	Embryo/Larvae & Embryo/Larvae	**Locomotor behavior** ↑ Distance in the dark (24 h, 6 days exposure) ↓ Distance in the dark (6 days exposure) ↑ Distance in the light (6 days exposure)
Barreto et al.^118^	Fosetyl-al	0.02, 0.2, 2, 20, 200	120 h	Embryo/Larvae	**Locomotor behavior** ↓ Distance traveled = Swimming time ↑ Velocity ↑ Acceleration ↑ Absolut turn angle**Neurochemical outcomes** = ChE activity = CAT activity ↑ GST activity
Brenet et al.^119^	Bixafen	0.08, 0.2	96 h	Embryo/Larvae	**Locomotor behavior** ↓ Distance traveled
Fan et al.^120^	Carbendazim	0.52, 0.65, 0.82, 1.02, 1.28, 1.6	24 h	Embryo/Embryo	**Locomotor behavior** ↑ Spontaneous movements
Forner-Piquer et al.^64^	Boscalid & Captan & Thiophanate & Ziram	0.00001, 0.00005, 0.001, 0.01, 0.1, 1, 10	120 h	Embryo/Larvae	**Locomotor behavior** ↓ Distance traveled ↓ Velocity
Huang et al.^121^	Fenamidone	0.03, 0.3, 0.4, 0.6	144 h	Embryo/Larvae	**Locomotor behavior** = Distance traveled (light–dark test) ↓ Distance in the dark (visual motor response test) = Distance in the light **Anxiety/fear-related behavior** ** = **Frequency in the dark = Time in the dark
Leandro et al.^122^	Mancozeb	0.005, 0.01, 0.02	24 h & 68 h & 164 h	Embryo/Embryo & Embryo/Larvae	**Locomotor behavior** ↓ Spontaneous movements (28 hpf) ↑ Number of stimuli (72 hpf) ↓ Response to touch (72 hpf) ↑ Distance traveled (0.005 mg/L, 168 hpf) ↓ Distance traveled (0.02 mg/L, 168 hpf) ↑ Absolut turn angle (0.005 mg/L, 168 hpf)↓ Absolut turn angle (0.02 mg/L, 168 hpf) ↑ Immobile episodes (0.02 mg/L, 168 hpf)↓ Immobile episodes (0.005 mg/L, 168 hpf) ↑ Immobile time (0.02 mg/L, 168 hpf) ↓ Immobile time (0.005 mg/L, 168 hpf) **Anxiety/fear-related behavior** = Time in the periphery (168 hpf) ↑ Entries in the periphery (168 hpf) **Neurochemical outcomes** ↑ AChE activity (28 hpf) ↓ AChE activity (72 hpf) ↓ SOD activity (24 hpf) ↑ SOD activity (72 hpf) ↓ CAT activity (72 hpf) ↑ CAT activity (168 hpf) ↑ GST activity (72 hpf) ↓ GST activity (168 hpf) ↑ ROS levels (72, 168 hpf)
Li et al.^123^	Azoxystrobin & Kresoxim-methyl & Pyraclostrobin & Trifloxystrobin	0.02027 & 0.01567 & 0.01939 & 0.02042	5 days	Embryo/Larvae	**Locomotor behavior** ↑ Distance in the dark (kresoxim-methyl, pyraclostrobin, trifloxystrobin) ↑ Distance in the light (trifloxystrobin)**Neurochemical outcomes**↑ MDA levels (pyraclostrobin, trifloxystrobin) ↑ SOD activity (pyraclostrobin, trifloxystrobin) ↑ CAT activity (trifloxystrobin) ↑ ROS levels (pyraclostrobin, trifloxystrobin)
Lin et al.^124^	Fluxapyroxad	1.1, 1.2, 1.3, 1.4, 1.5, 1.6	24 h	Embryo/Embryo	**Locomotor behavior** ↑ Spontaneous movements **Neurochemical outcomes** ↑ MDA levels = SOD activity = CAT activity ↑ GPx activity (0.174 mg/L) ↓ GPx activity (0.694 mg/L)
Paredes-Zúñiga et al.^125^	Triadimefon	5, 15	3 days	Adult/Adult	**Locomotor behavior** ↑ Time in the drug-paired zone (5 mg/L) ↓ Time in the drug-paired zone (15 mg/L) ↑ Circling behavior (days 1, 2)
Pompermaier et al.^126^	Copper	0.105	48 h	Adult/Adult	**Locomotor behavior** = Distance traveled = Absolut turn angle = Crossings **Anxiety/fear-related behavior** = Time in the upper zone = Time in the middle zone = Time in the bottom zone
Qian et al.^127^	Boscalid	0.3, 0.6, 1.2 & 0.01, 0.1, 1.0	8 days & 21 days	Embryo/Larvae & Adult/Adult	**Locomotor behavior** ↓ Distance traveled (larvae) ↑ Distance traveled (adult) ↓ Distance in the dark (larvae) ↓ Distance in the light (larvae) ↓ Velocity ↓ Acceleration ↓ Active time (larvae) ↑ Active time (larvae, adult) **Neurochemical outcomes** ↑ AChE levels (larvae) ↓ AChE activity (larvae)
Tang et al.^128^	Cyprodinil	0.0001, 0.001, 0.01	209–211 days & 215–217 days	Embryo/Adult	**Locomotor behavior** ↓ Distance traveled ↓ Velocity = Acceleration ↓ Absolut turn angle **Aggressive behavior** ↑ Time in the interaction zone
Wu et al.^129^	Procymidone	0.001, 0.01, 0.1	4 days & 7 days	Embryo/Larvae	**Locomotor behavior** ↑ Distance in the dark (4 days) ↑ Distance in the light (4 days) ↓ Distance in the dark (7 days) ↓ Distance in the light (7 days)
Yang^130^	Azoxystrobin & Pyraclostrobin & Trifloxystrobin	0.0002, 0.001, 0.005 & 0.77, 1.54, 2.32 & 0.51, 1, 2	6 days	Embryo/Larvae	**Locomotor behavior** ↑ Distance in the dark (azoxystrobin, pyraclostrobin) ↓ Distance in the dark (trifloxystrobin) = Distance in the light
Yang^131^	Thifluzamide	0.19, 1.9, 2.85	96 h & 144 h	Embryo/Larvae & Embryo/Larvae	**Locomotor behavior** ↓ Distance traveled ↓ Velocity ↓ Swimming activity ↓ Rotating frequency (144 hpf) **Neurochemical outcomes** ↓ AChE activity ↑ 5-HT levels ↑ Norepinephrine levels

The main findings were described as: ↑, higher when compared to the control group; ↓, lower when compared to the control group; = , no difference when compared to the control group. AChE = acetylcholinesterase, CAT = catalase, ChE = cholinesterase, DA = dopamine, GABA = gamma-aminobutyric acid, GPx = glutathione peroxidase, GSH = glutathione, GST = glutathione S-transferase, MDA = malondialdehyde, NPSH = non-protein thiols, ROS = reactive oxygen species, SH = thiols, SOD = superoxide dismutase, 5-HT = serotonin.

All the studies used immersion as the exposure method, whereas exposure durations ranged from 11 min to 217 days. The most recurrent duration of exposure among the publications was 24 h (n = 21, 35%), followed by 96 h (n = 12, 20%). It is important to emphasize that 24 h is usually employed to verify the outcome of spontaneous movements, while 96 h is recommended by the Organization for Economic Co-operation and Development (OECD) to assess acute fish toxicity in protocols 203 (adults) and 236 (embryos)^61,62^. Regarding the developmental stage during the exposure, the embryonic was the most common (n = 52, 86.7%). Subsequently, the larval stage was observed in 7 studies (11.6%) and the adult stage in 8 (13.3%). Some articles used more than one stage for the exposure.

The outcome assessment was mostly performed in larvae (n = 41, 68.33%) and embryos (n = 25, 41.7%). Some studies assessed the outcomes in more than one developmental stage.

The sex of the adult animals was mainly reported as an equal proportion of male and female (F:M), except for one in which it was not reported (unclear).

Regarding the authors included in this review, co-authorship network analysis identified 24 clusters of researchers investigating the neurobehavioral effects of fungicides globally (Fig. S1). An interactive version of the co-authorship network is available at https://tinyurl.com/239thp6t.

### Reporting quality

**Figure 2:**
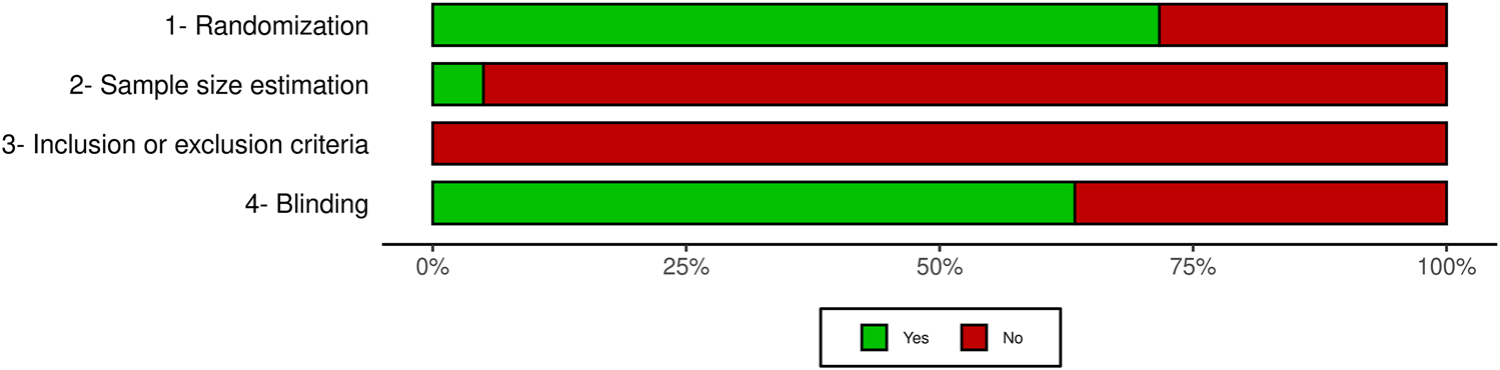
Reporting quality assessment of the included studies. The reporting quality assessment was performed by two independent investigators based on the criteria by^41^. Each item was scored as yes or no, meaning that the item is either reported or not, respectively. Classification is given as the percentage of assessed studies (n = 60) presenting each score.

The summary plot of the reporting quality evaluation is shown in Fig. 2. Randomization process was not cited in 17 studies (28.33%). Only 3 articles described methods for sample size estimation (5%), and none of the authors explicitly stated the data inclusion or exclusion criteria. Blinding was reported in 38 papers (63.33%). Individualized scores for each study included are available at https://osf.io/pgrhq.

### Meta-analysis

#### Distance

The meta-analysis included 61 comparisons from 12 independent studies. The total of animals used as controls was 1112, whereas the exposure individuals counted 2045. The highest concentration of fungicide in the meta-analysis was 20 mg/L for triadimefon^63^, while the lowest was 0.0001 mg/L for mepanipyrim, ziram, tiophanate, captan, and boscalid^26,64^.

**Figure 3:**
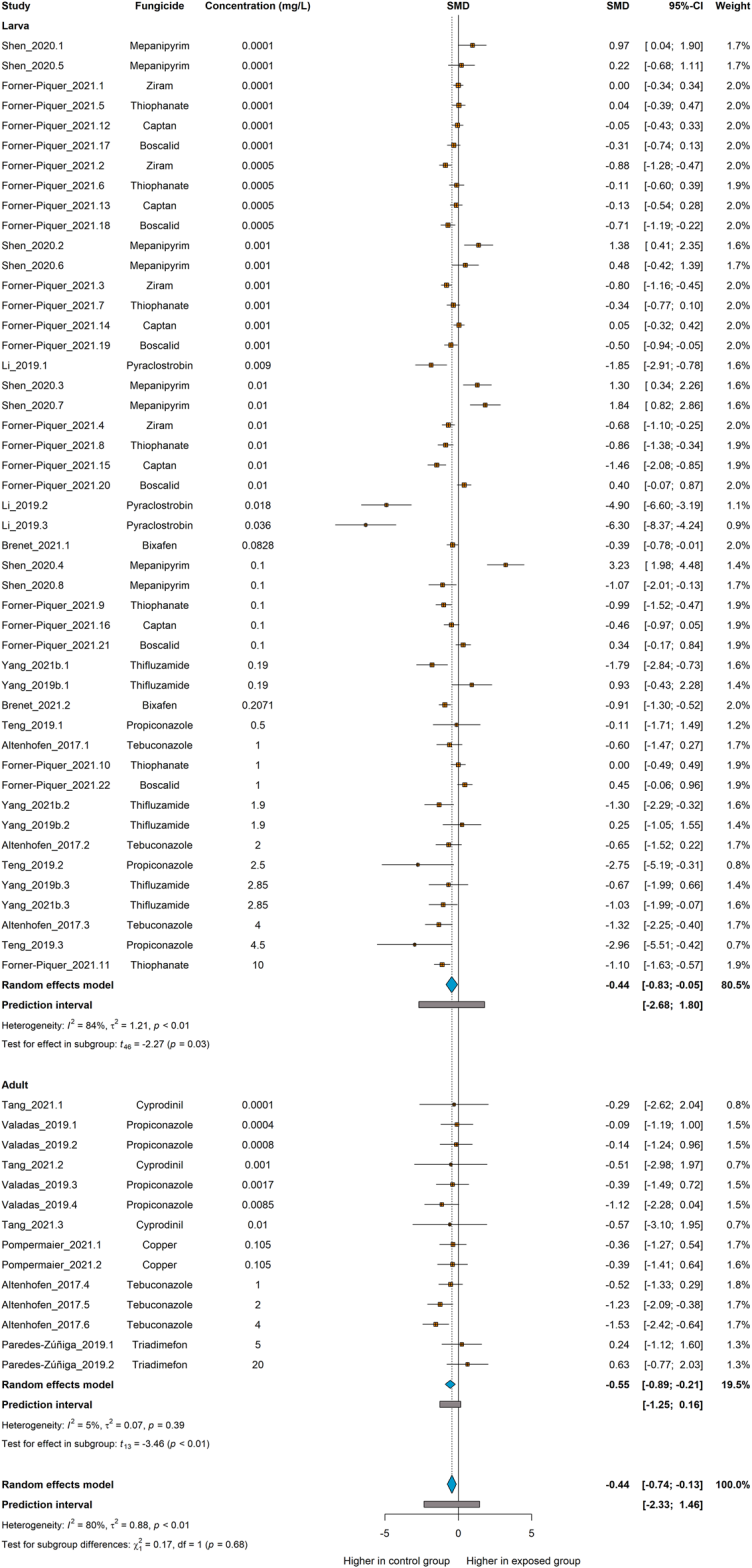
The effect of exposure to fungicides on distance traveled in zebrafish. Subgroup analyses were based on the developmental stage (either larva or adult). Data are presented as Hedges’ G standardized mean differences (SMD) and 95% confidence intervals.

The overall analysis showed that exposed animals present a lower distance traveled as compared to controls (SMD −0.44 [−0.74; −0.13], p = 0.0055, Fig. 3). The estimated heterogeneity was considered high, with an I^2^ = 80%, a τ^2^ = 0.88, and a Q = 300.1 (*df* = 60, p < 0.01). When calculating strictly for the developmental stage of the larvae, there was a significant effect of the fungicides on decreasing the distance traveled (SMD −0.44 [−0.83; −0.05], p = 0.03, Fig. 3). The heterogeneity was still considered high for this subgroup, with an I^2^ = 84%, a τ^2^ = 1.21, and a Q = 284.48 (p < 0.01). Similarly, analyzing the adults subgroup, there was a significant effect of the exposure to fungicides on decreasing the distance traveled (SMD −0.55 [−0.89; −0.21], p < 0.01, Fig. 3). Unlike the larvae, the heterogeneity was considered low, with an I^2^ = 5%, a τ^2^ = 0.07, and a Q = 13.72 (p = 0.39). The difference between subgroups was not significant (p = 0.68), indicating that the developmental stage is not a direct moderator for this outcome.

The result from the meta-analysis of distance using only fungicides of the triazole group was similar, and a decrease in distance was observed (Fig. S2).

#### Spontaneous movements

The meta-analysis comprised 64 comparisons from 13 independent studies. The total of embryos used as controls was 190, and the exposure individuals counted 670. The highest fungicide concentration in the meta-analysis was 145.89 mg/L for cyproconazole^65^, while the lowest was 0.0001 mg/L for cyprodinil^66^. All the experiments performed the outcome assessment at 24 h of exposure, except for one (48 h).

**Figure 4:**
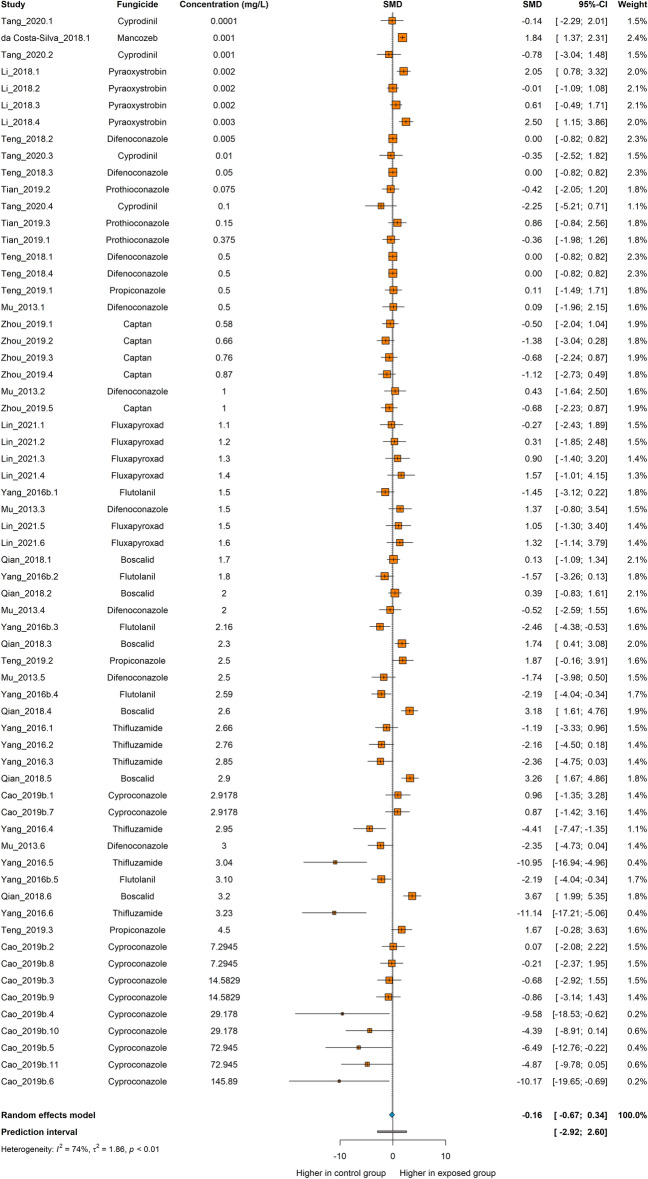
The effect of exposure to fungicides on spontaneous movements in zebrafish. Data are presented as Hedges’ G standardized mean differences (SMD) and 95% confidence intervals.

The overall analysis showed that fungicide exposure had no significant effect on the number of spontaneous movements (SMD −0.16 [−0.67; 0.34], p = 0.5265, Fig. 4). The estimated heterogeneity was considered moderate, with an I^2^ = 74%, a τ^2^ = 1.86, and a Q = 243.19 (*df* = 63, p < 0.01).

The result from the meta-analysis of spontaneous movements using only fungicides of the anilide or triazole groups was similar, and no significant effects were observed (Fig. S3 and S4, respectively).

The meta-regression of both outcomes showed no significant correlation of the concentration with the effects (Fig. S5 and S6). Meta-regressions excluding studies from^67^ (distance)^65^, and^68^ (spontaneous movements), maintained no significant correlation (Fig. S7 and S8).

Additional information regarding the meta-analysis can be found at https://osf.io/hdu5c/.

#### Publication bias

**Figure 5:**
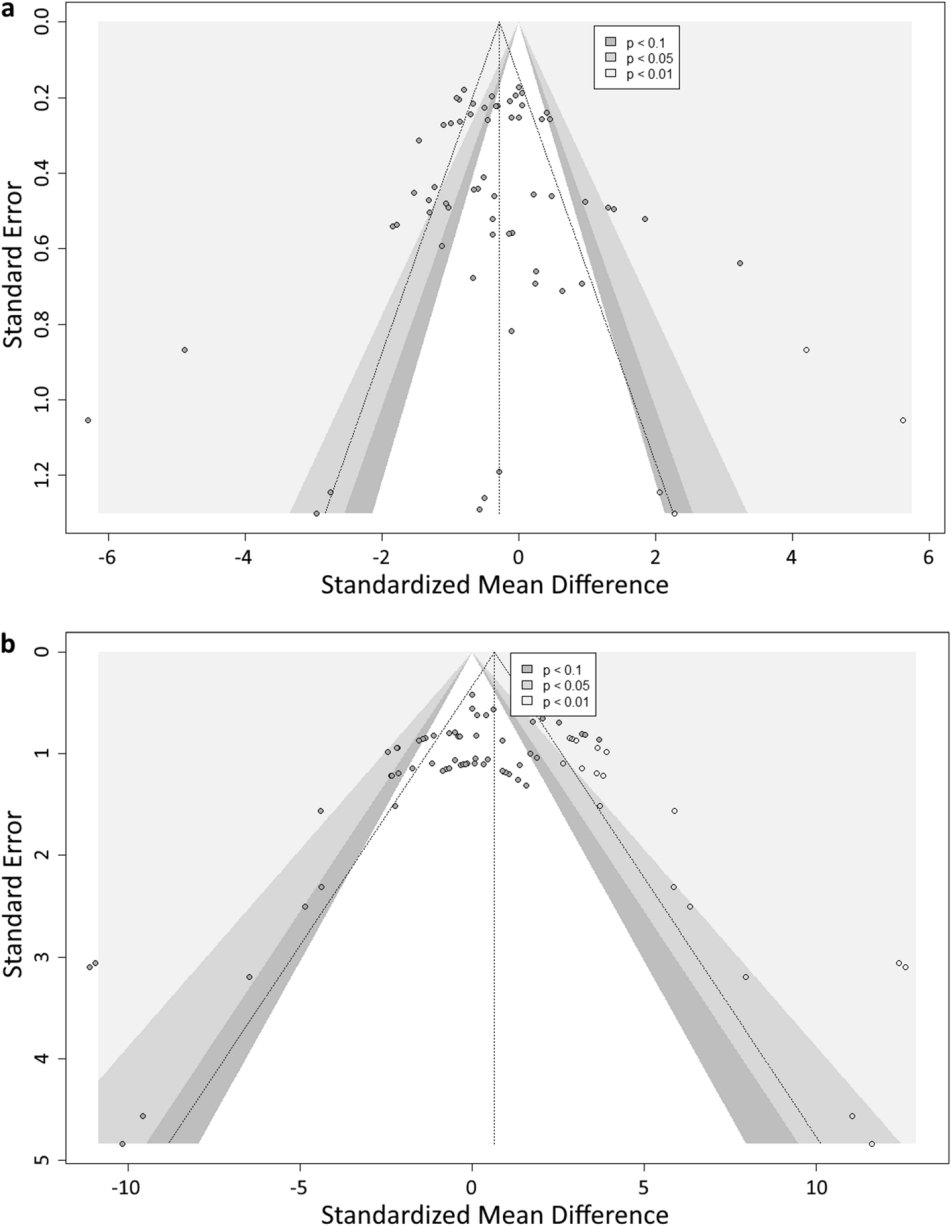
Funnel plot including studies analyzed within distance (**a**) and spontaneous movements (**b**) outcomes. Each gray circle represents a single comparison. Hollow circles represent imputed studies in the trim and fill analysis. The vertical line represents the overall effect size, and the triangular region represents the 95% confidence interval. Shaded areas represent the interval for statistically significant effects.

Visual inspection of the funnel plot for the distance outcome showed an asymmetrical distribution of the studies (Fig. 5a). Trim and fill analysis for distance imputed 4 studies to the meta-analysis. The overall effect of the fungicide exposure was no longer significant for this outcome when imputing potentially unpublished data (SMD −0.29 [−0.66, 0.08], p = 0.1252).

For spontaneous movements, the funnel plot also demonstrated an asymmetrical distribution (Fig. 5b). Trim and fill analysis for this outcome imputed 20 studies to the meta-analysis, and the overall effect of fungicide exposure remained not significant (SMD 0.64 [−0.02, 1.29], p = 0.0568).

Egger’s regression test indicated publication bias only for spontaneous movements, which showed a p < 0.0001 (for distance, p = 0.4120) (Table S1).

#### Sensitivity analysis

**Figure 6:**
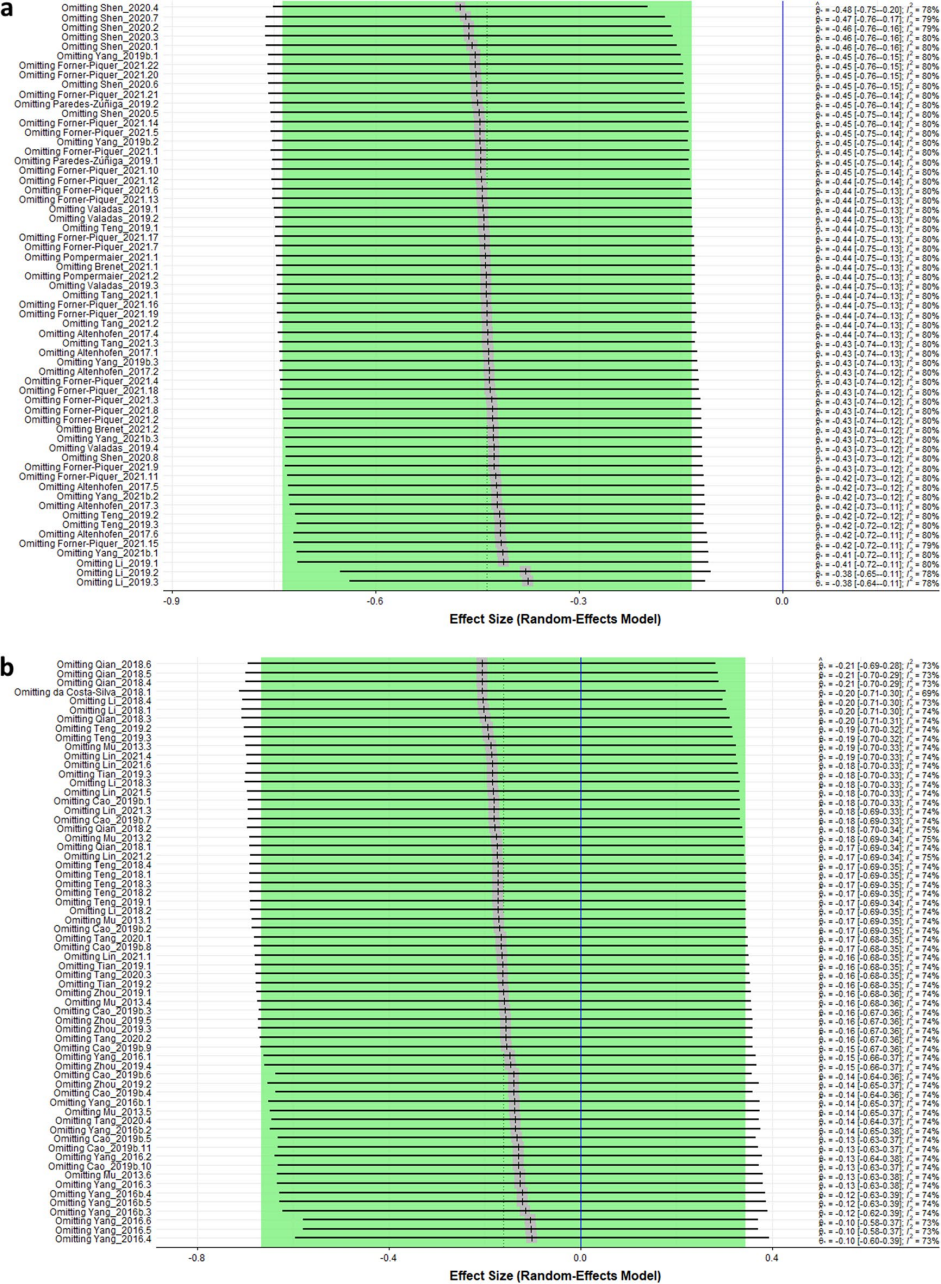
Sensitivity analyses for studies for distance (**a**) and spontaneous movements (**b**) outcomes. Data are presented as Hedges’ G standardized mean differences (SMD) and 95% confidence intervals.

The leave-one-out analysis for distance revealed that none of the comparisons significantly modified the meta-analysis result (Fig. 6a). The overall effect and heterogeneity remained close to the original value. However, to confirm that any isolated study is skewing the results, we performed another meta-analysis, excluding all the comparisons from the study by^67^. This study showed unusually high SMD in the forest plot, and the omission of their experiments in the leave-one-out analysis altered the overall effect direction. The significant overall effect was sustained (SMD −0.31 [−0.54; −0.08] (Fig. S9).

The leave-on-out analysis for spontaneous movements showed that omitting comparisons did not significantly modify the meta-analysis original result (Fig. 6b). We also ran the meta-analysis without 2 studies:^65,68^. In the forest plot, these studies showed atypically high SMD, and omitting their experiments in the leave-one-out analysis changed the overall effect direction. Although the direction of the effect changed, it was still not significant (SMD 0.22 [−021; 066]) (Fig. S10).

## Discussion

This work aimed to evaluate and synthesize the neurobehavioral effects of fungicide exposure in zebrafish through a systematic review and meta-analysis. As main findings, we can highlight that fungicides cause a decrease in distance traveled by larval and adult zebrafish; no effect was observed on spontaneous movements of embryos.

The locomotor behavior was the category most frequently assessed in the included studies. Along with distance traveled, velocity was also commonly reported. It is important to emphasize that a decreased distance traveled or velocity does not necessarily imply toxicity, as a substance may have a sedative effect. However, even if not directly related to toxicity or locomotor damage, altered locomotion poses a risk to organisms as it impacts their ability to forage, reproduce, and escape predators^69^. These data should be observed together with the neurochemical outcomes, which were also consistently investigated and are linked to behavioral variation. The included studies frequently reported altered outcomes related to enzymatic activity, some involved in locomotion (AChE) and oxidative status (GST, SOD, GPx, among others), which are possible mechanisms for reduced locomotor behavior. Few included studies reported investigations of behavioral domains other than locomotor (9), and even so, it was limited to anxiety-fear-related and aggressive behavior, revealing a gap in the literature. The lack of standardized protocols or unpublished negative results could explain this observation^70^.

The overall high heterogeneity observed in the meta-analysis for distance traveled can be attributed to several sources. The experimental conditions, from rearing until exposure and tests, are extremely variable between laboratories. The researchers employed many protocols, including distinct durations of exposure, frequency of solution renewal, number of coexposed animals, age of the fish, type, and test apparatus. When considering the subgroup analysis, studies with adults had a lower heterogeneity than those performed at the larval stage. Even though fewer adult studies were included, we can indeed verify more uniformity between the protocols of these experiments, mostly during the outcome assessment. Therefore, this similarity can explain the low heterogeneity of this subgroup.

Interestingly, there was no significant difference between the subgroups, indicating that the developmental stage of the animals does not significantly impact the effect of fungicides on the distance traveled. Despite the different locomotor mechanisms exhibited by adults and larvae^69^, it suggests that fungicide exposure consistently affects both subgroups.

On the other hand, the heterogeneity of the outcome of spontaneous movements was considered moderate. Unlike the distance traveled, the spontaneous movements can be measured in a single developmental stage: the embryo, generally at 24 h post-fertilization (hpf). Consequently, the age of the animals can be excluded as a potential source of heterogeneity, which helps to explain why the heterogeneity did not reach the highest level.

The reporting quality analysis showed a high percentage of negative answers, especially regarding “sample size estimation” and “inclusion or exclusion criteria”. None of the authors explicitly stated previously determined parameters for the eligibility of the data. The result from this evaluation indicates that the conclusions of this review should be interpreted with caution since the report of the included studies presents considerable uncertainty. This lack of methodological information has been recognized as one of the main reasons behind the reproducibility crisis in preclinical research^71^. Aiming to improve the quality of the studies, guidelines for the research report with animals have been developed in the last years^72^; however, it is a multifaceted problem that demands complex and long-term solutions^73^.

Trim and fill analysis for distance imputed 4 studies into the meta-analysis, resulting in no overall significant effect. This fact suggests the presence of missing studies with null and/or significant results^74^. The unpublished data may have influenced the previously observed significant effect, revealing a potential bias towards the publication of studies only with significant findings in which fungicide exposure decreases locomotion. However, Egger's test suggests no evidence of publication bias.

Despite the input of 20 studies in the trim and fill analysis for spontaneous movements, it did not alter the non-significant overall effect found in the meta-analysis. This indicates that publication bias may not explain the observed non-significance. However, it is important to note that the significant result obtained from Egger's test indicates the presence of potential publication bias. The Egger's test suggests a tendency to publish studies with significant results, which could skew the meta-analysis. Although the trim and fill analysis did not change the overall effect, the imputed studies may impact the precision and confidence interval of the effect estimate. There is an important role of selective publishing in the misinterpretation of a meta-analysis^75^, highlighting the need for new practices regarding the publication of non-significant results. Even if this represents a complex, deep-rooted issue that requires a change in the whole culture of publishing scientific data, some authors have been raising this discussion and proposing alternatives^76–79^. However, the results of our publication bias analysis should be interpreted with caution, as our funnel plots were based on SMD versus standard error (SE). Although this method is standard practice in the field, it may introduce distortion and overestimate the existence of publication bias, as demonstrated empirically by Zwetsloot et al.^80^.

The sensitivity analysis indicated that the meta-analysis results were not significantly influenced by any particular study or set of studies, suggesting that the overall effect size is robust and reliable. This finding supports the validity of the meta-analytic conclusions and can increase the confidence in the reliability of the results. However, the reliability of each comparison could not be determined due to poor reporting practices and a general lack of protocol preregistration.

One limitation of this study was the inclusion of only studies that used analytical-grade fungicides while excluding those involving commercial formulations and fungicide mixtures. This exclusion was necessary to isolate the specific effects of individual chemicals and ensure more accurate conclusions. Although this approach may be less realistic, it enhances the precision of the findings. Additionally, we did not restrict the inclusion criteria to studies involving exposure to environmentally relevant concentrations, as this would severely reduce the number of eligible articles, making it impossible to conduct a comprehensive meta-analysis. Another significant limitation worth highlighting is the potential inclusion of fraudulent data, which becomes evident when implausible results are observed. While various tools and techniques exist to perform statistical checks and verify data integrity, it is important to note that there is currently no foolproof method to confirm whether a study is fraudulent or not definitively. This task becomes even more challenging without direct access to the data.

Our results reinforce the effects of these chemicals, with their misuse representing a threat to the ecosystems. Since we depend on the affected environment, its contamination is an alert to public health. Besides that, we confirm the demand for well-designed studies with greater clarity of report on this topic. The authors should clearly state key elements such as sample size, sample size estimation, data inclusion or exclusion criteria, and blinding. Some available tools, like preregistration of study protocols and adherence to animal studies reporting guidelines such as the ARRIVE^72^, could be useful. Compliance with specific reporting guidelines for ecotoxicological studies as the “Criteria for Reporting and evaluating Ecotoxicity Data” (CRED)^81^ is also highly encouraged. In addition, standardization of behavioral tests could enable more comprehensive meta-analyses. These recommendations can lead to more reliable conclusions and contribute to effectively monitoring environmental pollution.

### Supplementary Information

Supplementary Information.

## Data Availability

All data are available at Open Science Framework (https://osf.io/hdu5c/).
