# Supplementary material for: Neurobehavioral effects of fungicides in zebrafish: a systematic review and meta-analysis
Source: Sci Rep. 2023 Oct 24;13:18142. doi: 10.1038/s41598-023-45350-6 (PMC10598008; doi:10.1038/s41598-023-45350-6)
Supplement: Supplementary file 1 — Supplementary Information. [file 41598_2023_45350_MOESM1_ESM.pdf]

Supplementary Information

Neurobehavioral effects of fungicides in zebrafish: a systematic review and meta-analysis

Carlos G. Reis, Leonardo M. Bastos, Rafael Chitolina, Matheus Gallas-Lopes, Querusche K. Zanona, Sofia Z. Becker, Ana P. Herrmann, Angelo Piato

==

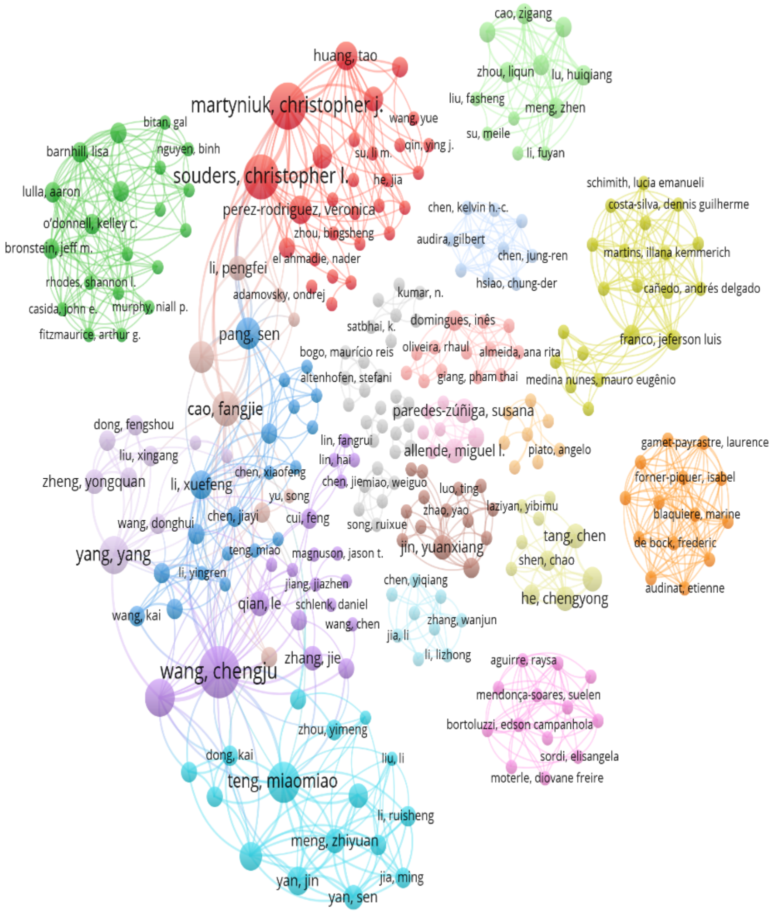

==

Fig. S1. Co-authorship network analysis of researchers who authored the included articles investigating neurobehavioral effects of fungicides in zebrafish. The size of the circles represents the number of studies published by each author. The color of the

lines and circles differentiates the clusters of authors. The distance between the two circles indicates the correlations between researchers.

==

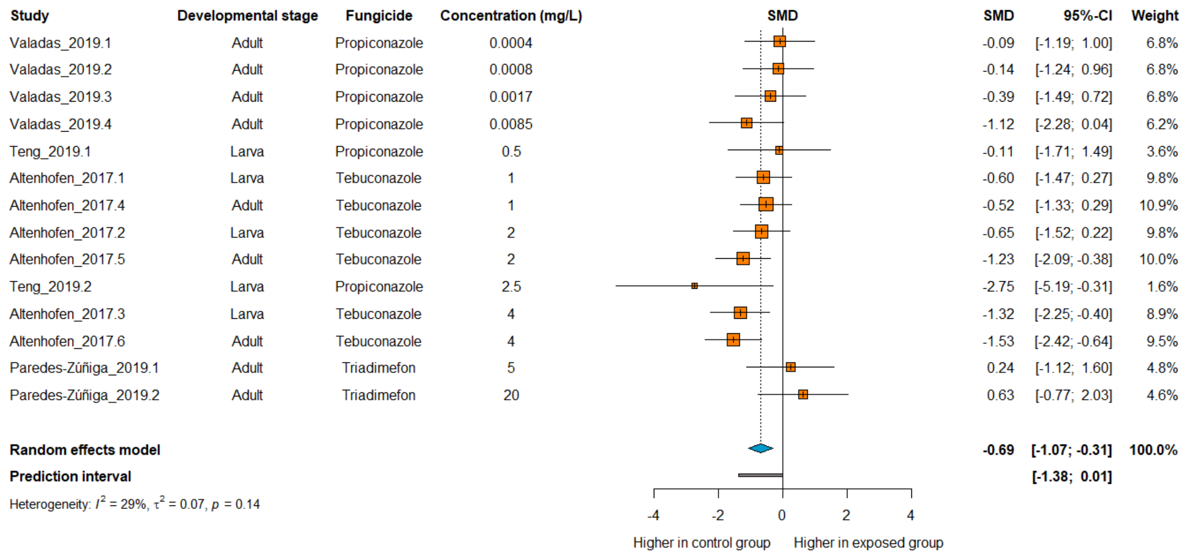

==

Fig. S2. The effect of exposure to triazole fungicides on distance traveled in zebrafish. Data are presented as Hedges' G standardized mean differences (SMD) and 95% confidence intervals.

==

Study	Developmental stage	Fungicide	Concentration (mg/L)	SMD	95%-CI	Weight
Lin_2021.1	Embryo	Fluxapyroxad	1.1	-0.27	[-2.43; 1.89]	4.5%
Lin_2021.2	Embryo	Fluxapyroxad	1.2	0.31	[-1.85; 2.48]	4.4%
Lin_2021.3	Embryo	Fluxapyroxad	1.3	0.90	[-1.40; 3.20]	4.4%
Lin_2021.4	Embryo	Fluxapyroxad	1.4	1.57	[-1.01; 4.15]	4.1%
Yang_2016b.1	Embryo	Flutolanil	1.5	-1.45	[-3.12; 0.22]	4.8%
Lin_2021.5	Embryo	Fluxapyroxad	1.5	1.05	[-1.30; 3.40]	4.3%
Lin_2021.6	Embryo	Fluxapyroxad	1.6	1.32	[-1.14; 3.79]	4.2%
Qian_2018.1	Embryo	Boscalid	1.7	0.13	[-1.09; 1.34]	5.1%
Yang_2016b.2	Embryo	Flutolanil	1.8	-1.57	[-3.26; 0.13]	4.8%
Qian_2018.2	Embryo	Boscalid	2	0.39	[-0.83; 1.61]	5.1%
Yang_2016b.3	Embryo	Flutolanil	2.16	-2.46	[-4.38; -0.53]	4.6%
Qian_2018.3	Embryo	Boscalid	2.3	1.74	[0.41; 3.08]	5.0%
Yang_2016b.4	Embryo	Flutolanil	2.59	-2.19	[-4.04; -0.34]	4.7%
Qian_2018.4	Embryo	Boscalid	2.6	3.18	[1.61; 4.76]	4.9%
Yang_2016.1	Embryo	Thifluzamide	2.66	-1.19	[-3.33; 0.96]	4.5%
Yang_2016.2	Embryo	Thifluzamide	2.76	-2.16	[-4.50; 0.18]	4.3%
Yang_2016.3	Embryo	Thifluzamide	2.85	-2.36	[-4.75; 0.03]	4.3%
Qian_2018.5	Embryo	Boscalid	2.9	3.26	[1.67; 4.86]	4.8%
Yang_2016.4	Embryo	Thifluzamide	2.95	-4.41	[-7.47; -1.35]	3.8%
Yang_2016.5	Embryo	Thifluzamide	3.04	-10.95	[-16.94; -4.96]	2.1%
Yang_2016b.5	Embryo	Flutolanil	3.1	-2.19	[-4.04; -0.34]	4.7%
Qian_2018.6	Embryo	Boscalid	3.2	3.67	[1.99; 5.35]	4.8%
Yang_2016.6	Embryo	Thifluzamide	3.23	-11.14	[-17.21; -5.06]	2.0%
**Random effects model**				**-0.52**	**[-1.84; 0.81]**	**100.0%**
**Prediction interval**					**[-5.63; 4.60]**	

Heterogeneity: $I^2 = 83\%$, $\tau^2 = 5.74$, $p < 0.01$

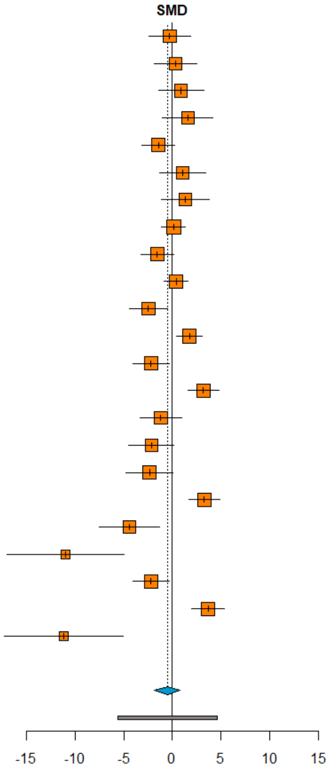

==

Fig. S3. The effect of exposure to anilide fungicides on spontaneous movements in zebrafish. Data are presented as Hedges' G standardized mean differences (SMD) and 95% confidence intervals.

==

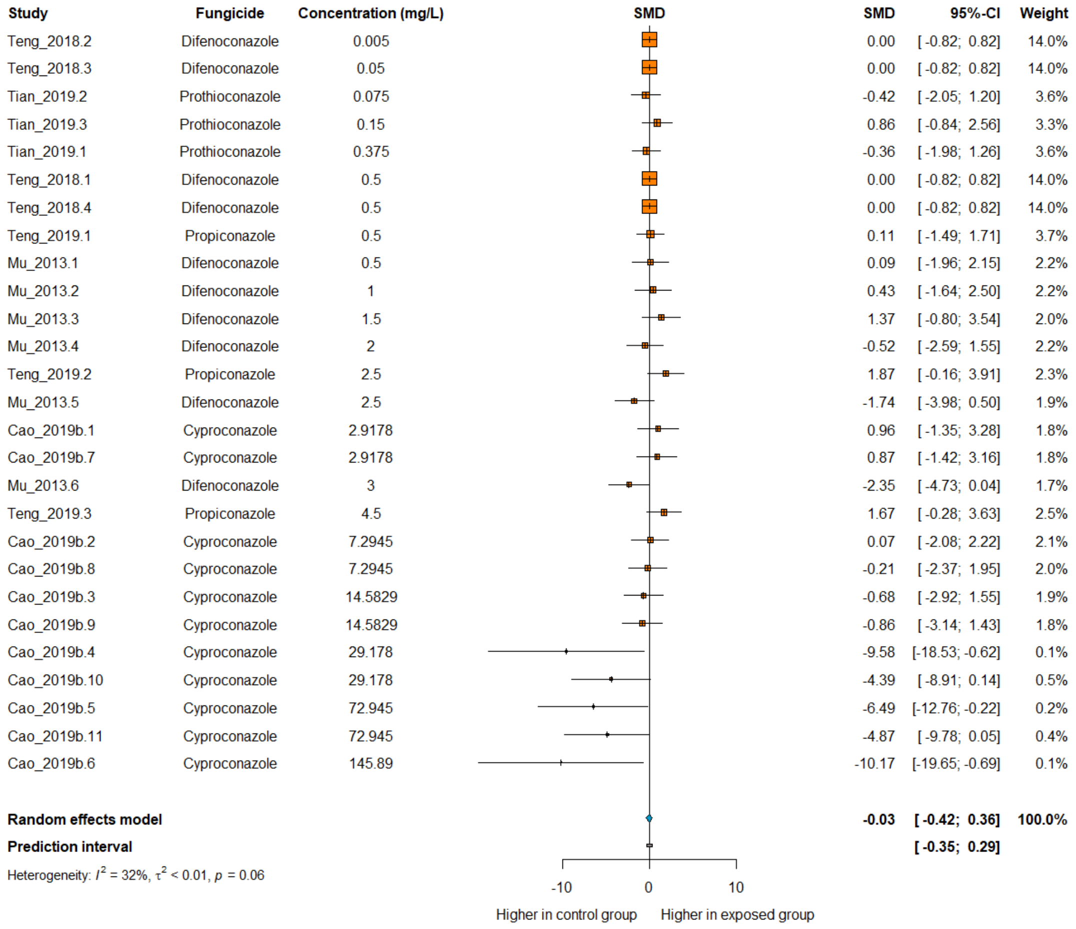

==

Fig. S4. The effect of exposure to triazole fungicides on spontaneous movements in zebrafish. Data are presented as Hedges' G standardized mean differences (SMD) and 95% confidence intervals.

==

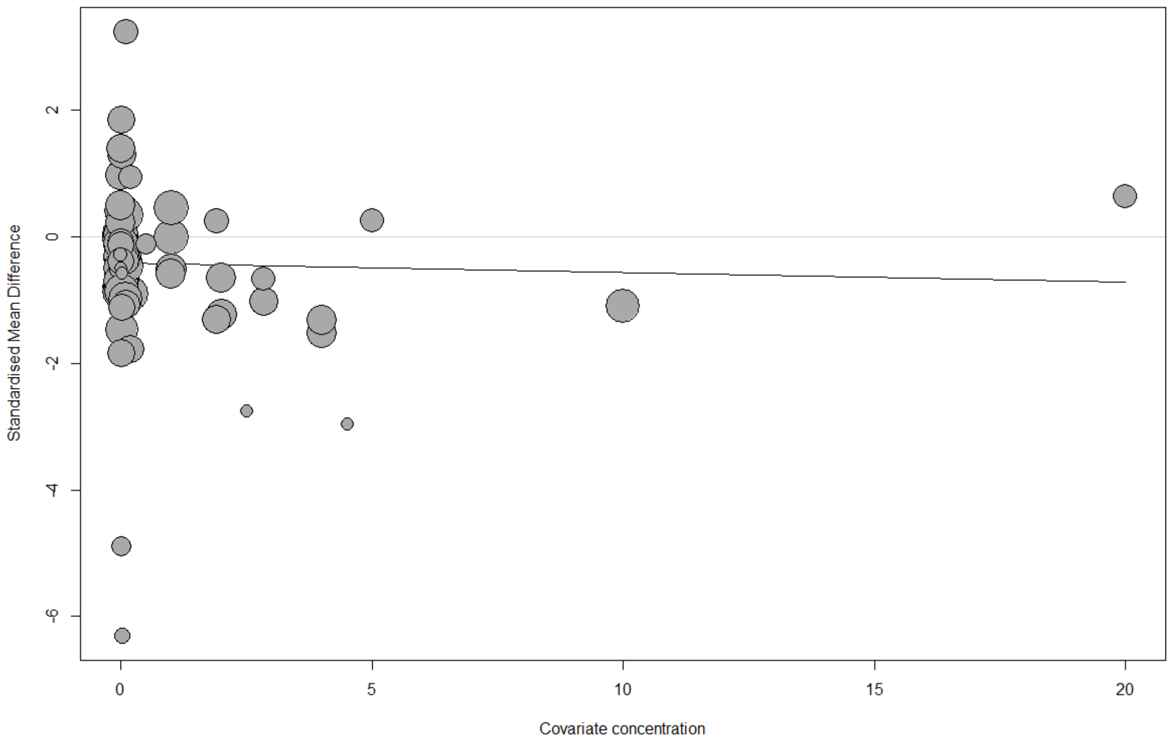

==

Fig. S5. Meta-regression of distance using the concentration of fungicides as the moderator variable.

==

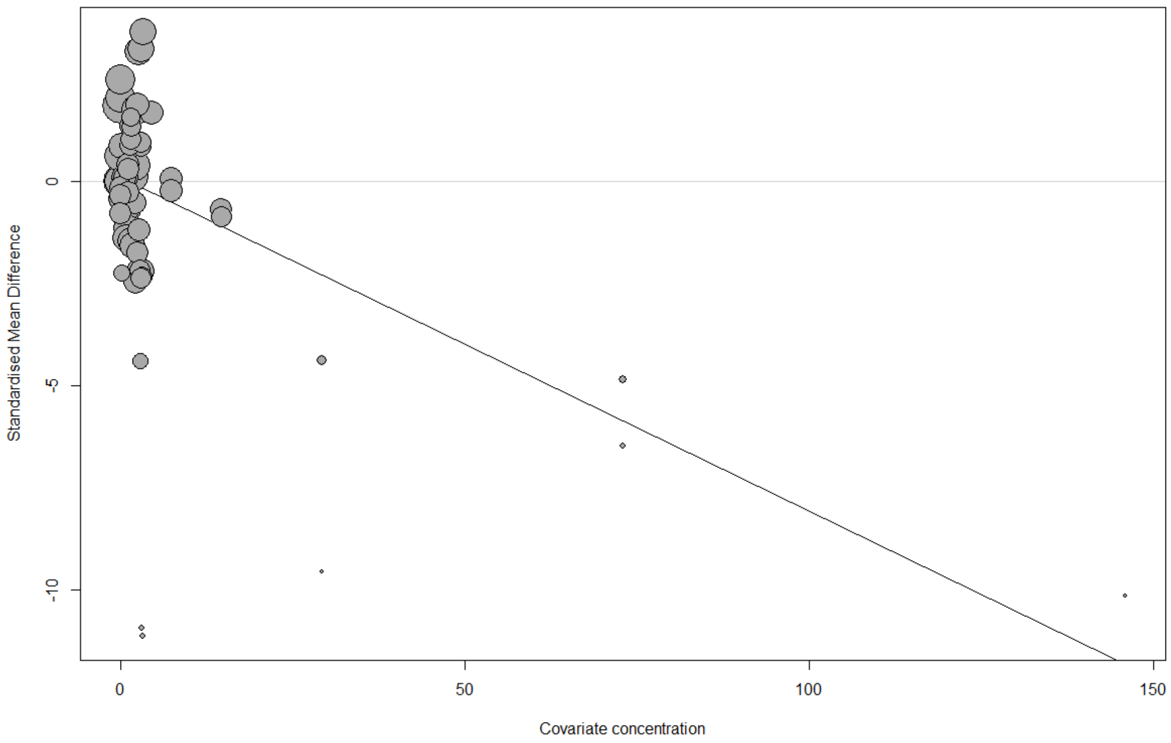

==

Fig. S6. Meta-regression of spontaneous movements using the concentration of fungicides as the moderator variable.

==

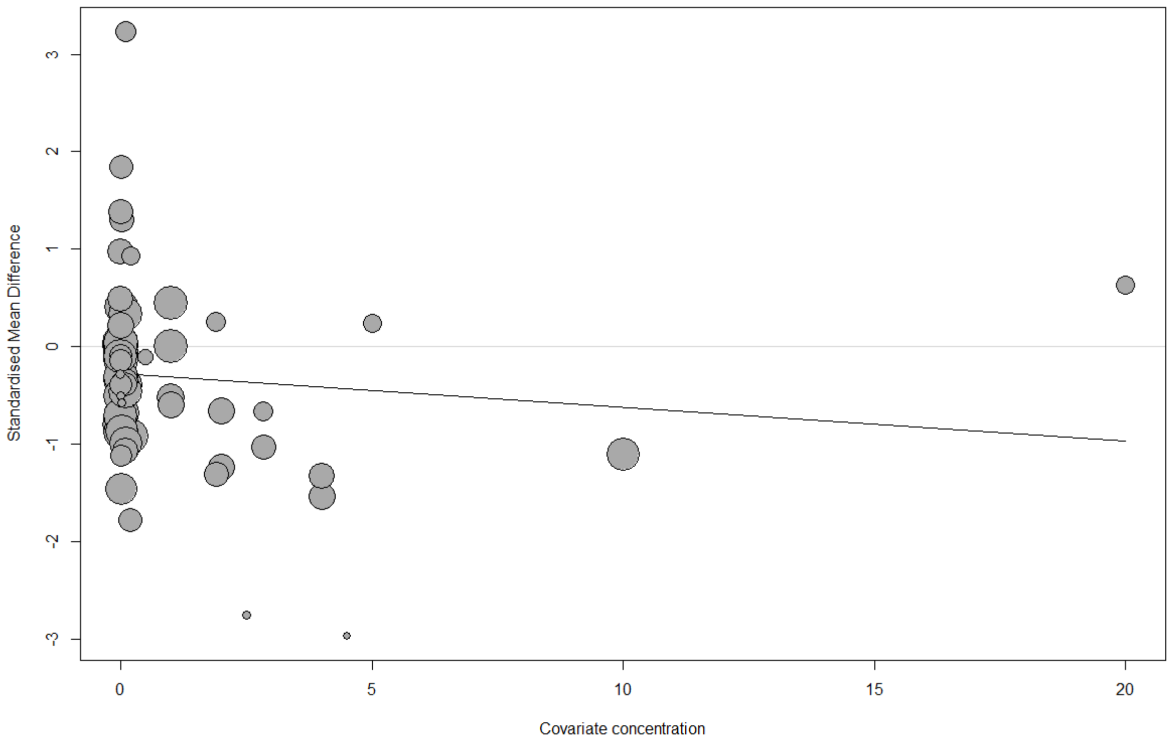

==

Fig. S7. Meta-regression of distance excluding the study by [67] using fungicide concentration as the moderator variable.

==

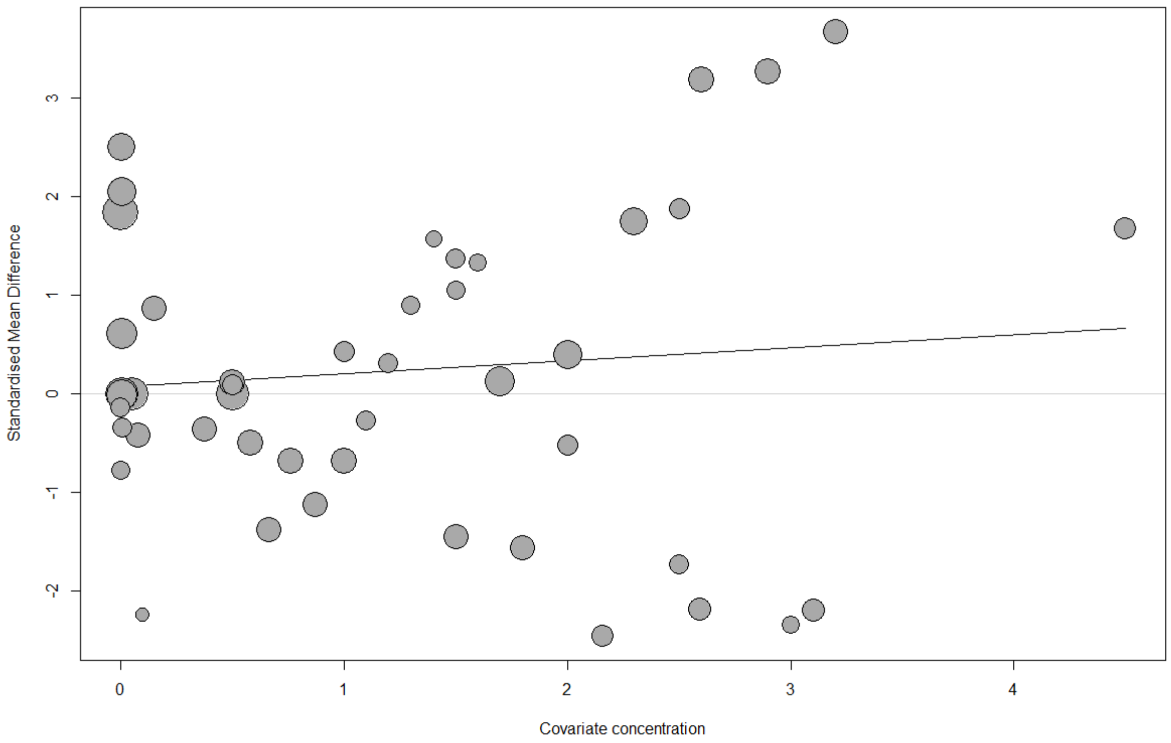

==

Fig. S8. Meta-regression of spontaneous movements excluding the studies by [65] and [68] using fungicide concentration as the moderator variable.

Table S1. Egger’s regression test summary for the outcomes of distance and spontaneous movements.

===

Outcome	Intercept	Standard error	t	*p*-value
Distance	-0.2221	0.1990	-0.83	0.4120
Spontaneous movements	1.8723	0.3385	-5.10	< 0.0001

===

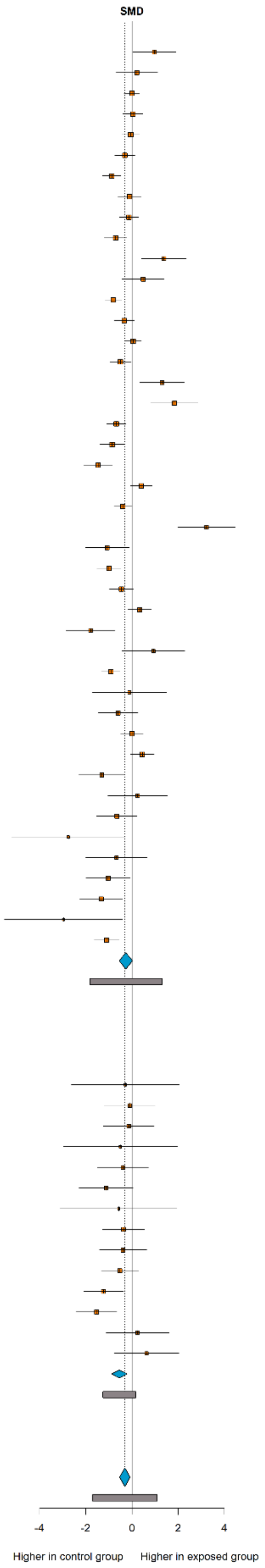

Study	Fungicide	Concentration (mg/L)	SMD	95%-CI	Weight
**Larva**					
Shen_2020.1	Mepanipyrim	0.0001	0.97	[0.04; 1.90]	1.7%
Shen_2020.5	Mepanipyrim	0.0001	0.22	[-0.68; 1.11]	1.7%
Forner-Piquer_2021.1	Ziram	0.0001	0.00	[-0.34; 0.34]	2.3%
Forner-Piquer_2021.5	Thiophanate	0.0001	0.04	[-0.39; 0.47]	2.2%
Forner-Piquer_2021.12	Captan	0.0001	-0.05	[-0.43; 0.33]	2.3%
Forner-Piquer_2021.17	Boscalid	0.0001	-0.31	[-0.74; 0.13]	2.2%
Forner-Piquer_2021.2	Ziram	0.0005	-0.88	[-1.28; -0.47]	2.3%
Forner-Piquer_2021.6	Thiophanate	0.0005	-0.11	[-0.60; 0.39]	2.2%
Forner-Piquer_2021.13	Captan	0.0005	-0.13	[-0.54; 0.28]	2.2%
Forner-Piquer_2021.18	Boscalid	0.0005	-0.71	[-1.19; -0.22]	2.2%
Shen_2020.2	Mepanipyrim	0.001	1.38	[0.41; 2.35]	1.6%
Shen_2020.6	Mepanipyrim	0.001	0.48	[-0.42; 1.39]	1.7%
Forner-Piquer_2021.3	Ziram	0.001	-0.80	[-1.16; -0.45]	2.3%
Forner-Piquer_2021.7	Thiophanate	0.001	-0.34	[-0.77; 0.10]	2.2%
Forner-Piquer_2021.14	Captan	0.001	0.05	[-0.32; 0.42]	2.3%
Forner-Piquer_2021.19	Boscalid	0.001	-0.50	[-0.94; -0.05]	2.2%
Shen_2020.3	Mepanipyrim	0.01	1.30	[0.34; 2.26]	1.6%
Shen_2020.7	Mepanipyrim	0.01	1.84	[0.82; 2.86]	1.6%
Forner-Piquer_2021.4	Ziram	0.01	-0.68	[-1.10; -0.25]	2.2%
Forner-Piquer_2021.8	Thiophanate	0.01	-0.86	[-1.38; -0.34]	2.1%
Forner-Piquer_2021.15	Captan	0.01	-1.46	[-2.08; -0.85]	2.0%
Forner-Piquer_2021.20	Boscalid	0.01	0.40	[-0.07; 0.87]	2.2%
Brenet_2021.1	Bixafen	0.0828	-0.39	[-0.78; -0.01]	2.3%
Shen_2020.4	Mepanipyrim	0.1	3.23	[1.98; 4.48]	1.3%
Shen_2020.8	Mepanipyrim	0.1	-1.07	[-2.01; -0.13]	1.7%
Forner-Piquer_2021.9	Thiophanate	0.1	-0.99	[-1.52; -0.47]	2.1%
Forner-Piquer_2021.16	Captan	0.1	-0.46	[-0.97; 0.05]	2.1%
Forner-Piquer_2021.21	Boscalid	0.1	0.34	[-0.17; 0.84]	2.2%
Yang_2021b.1	Thifluzamide	0.19	-1.79	[-2.84; -0.73]	1.5%
Yang_2019b.1	Thifluzamide	0.19	0.93	[-0.43; 2.28]	1.2%
Brenet_2021.2	Bixafen	0.2071	-0.91	[-1.30; -0.52]	2.3%
Teng_2019.1	Propiconazole	0.5	-0.11	[-1.71; 1.49]	1.0%
Altenhofen_2017.1	Tebuconazole	1	-0.60	[-1.47; 0.27]	1.7%
Forner-Piquer_2021.10	Thiophanate	1	0.00	[-0.49; 0.49]	2.2%
Forner-Piquer_2021.22	Boscalid	1	0.45	[-0.06; 0.96]	2.2%
Yang_2021b.2	Thifluzamide	1.9	-1.30	[-2.29; -0.32]	1.6%
Yang_2019b.2	Thifluzamide	1.9	0.25	[-1.05; 1.55]	1.3%
Altenhofen_2017.2	Tebuconazole	2	-0.65	[-1.52; 0.22]	1.7%
Teng_2019.2	Propiconazole	2.5	-2.75	[-5.19; -0.31]	0.6%
Yang_2019b.3	Thifluzamide	2.85	-0.67	[-1.99; 0.66]	1.2%
Yang_2021b.3	Thifluzamide	2.85	-1.03	[-1.99; -0.07]	1.6%
Altenhofen_2017.3	Tebuconazole	4	-1.32	[-2.25; -0.40]	1.7%
Teng_2019.3	Propiconazole	4.5	-2.96	[-5.51; -0.42]	0.5%
Forner-Piquer_2021.11	Thiophanate	10	-1.10	[-1.63; -0.57]	2.1%
**Random effects model**			**-0.26**	**[-0.55; 0.02]**	**81.5%**
**Prediction interval**				**[-1.82; 1.30]**	
Heterogeneity: $I^2 = 80\%$, $\tau^2 = 0.58$, $p < 0.01$					
Test for effect in subgroup: $t_{43} = -1.84$ ($p = 0.07$)					
**Adult**					
Tang_2021.1	Cyprodinil	0.0001	-0.29	[-2.62; 2.04]	0.6%
Valadas_2019.1	Propiconazole	0.0004	-0.09	[-1.19; 1.00]	1.5%
Valadas_2019.2	Propiconazole	0.0008	-0.14	[-1.24; 0.96]	1.5%
Tang_2021.2	Cyprodinil	0.001	-0.51	[-2.98; 1.97]	0.6%
Valadas_2019.3	Propiconazole	0.0017	-0.39	[-1.49; 0.72]	1.5%
Valadas_2019.4	Propiconazole	0.0085	-1.12	[-2.28; 0.04]	1.4%
Tang_2021.3	Cyprodinil	0.01	-0.57	[-3.10; 1.95]	0.5%
Pompermaier_2021.1	Copper	0.105	-0.36	[-1.27; 0.54]	1.7%
Pompermaier_2021.2	Copper	0.105	-0.39	[-1.41; 0.64]	1.6%
Altenhofen_2017.4	Tebuconazole	1	-0.52	[-1.33; 0.29]	1.8%
Altenhofen_2017.5	Tebuconazole	2	-1.23	[-2.09; -0.38]	1.7%
Altenhofen_2017.6	Tebuconazole	4	-1.53	[-2.42; -0.64]	1.7%
Paredes-Zúñiga_2019.1	Triadimefon	5	0.24	[-1.12; 1.60]	1.2%
Paredes-Zúñiga_2019.2	Triadimefon	20	0.63	[-0.77; 2.03]	1.2%
**Random effects model**			**-0.55**	**[-0.89; -0.21]**	**18.5%**
**Prediction interval**				**[-1.25; 0.16]**	
Heterogeneity: $I^2 = 5\%$, $\tau^2 = 0.07$, $p = 0.39$					
Test for effect in subgroup: $t_{13} = -3.46$ ($p < 0.01$)					
**Random effects model**			**-0.31**	**[-0.54; -0.08]**	**100.0%**
**Prediction interval**				**[-1.70; 1.09]**	
Heterogeneity: $I^2 = 76\%$, $\tau^2 = 0.48$, $p < 0.01$					
Test for subgroup differences: $\chi^2_1 = 1.80$, df = 1 ($p = 0.18$)					

==

Fig. S9. The effect of exposure to fungicides on distance traveled in zebrafish excluding the study by [67]. Data are presented as Hedges' G standardized mean differences (SMD) and 95% confidence intervals.

Study	Fungicide	Concentration (mg/L)	SMD	95%-CI	Weight
Tang_2020.1	Cyprodinil	0.0001	-0.14	[-2.29; 2.01]	1.8%
da Costa-Silva_2018.1	Mancozeb	0.001	1.84	[1.37; 2.31]	3.1%
Tang_2020.2	Cyprodinil	0.001	-0.78	[-3.04; 1.48]	1.7%
Li_2018.1	Pyraoxystrobin	0.002	2.05	[0.78; 3.32]	2.5%
Li_2018.2	Pyraoxystrobin	0.002	-0.01	[-1.09; 1.08]	2.7%
Li_2018.3	Pyraoxystrobin	0.002	0.61	[-0.49; 1.71]	2.7%
Li_2018.4	Pyraoxystrobin	0.003	2.50	[1.15; 3.86]	2.4%
Teng_2018.2	Difenoconazole	0.005	0.00	[-0.82; 0.82]	2.9%
Tang_2020.3	Cyprodinil	0.01	-0.35	[-2.52; 1.82]	1.8%
Teng_2018.3	Difenoconazole	0.05	0.00	[-0.82; 0.82]	2.9%
Tian_2019.2	Prothioconazole	0.075	-0.42	[-2.05; 1.20]	2.2%
Tang_2020.4	Cyprodinil	0.1	-2.25	[-5.21; 0.71]	1.3%
Tian_2019.3	Prothioconazole	0.15	0.86	[-0.84; 2.56]	2.1%
Tian_2019.1	Prothioconazole	0.375	-0.36	[-1.98; 1.26]	2.2%
Teng_2018.1	Difenoconazole	0.5	0.00	[-0.82; 0.82]	2.9%
Teng_2018.4	Difenoconazole	0.5	0.00	[-0.82; 0.82]	2.9%
Teng_2019.1	Propiconazole	0.5	0.11	[-1.49; 1.71]	2.2%
Mu_2013.1	Difenoconazole	0.5	0.09	[-1.96; 2.15]	1.8%
Zhou_2019.1	Captan	0.58	-0.50	[-2.04; 1.04]	2.3%
Zhou_2019.2	Captan	0.66	-1.38	[-3.04; 0.28]	2.2%
Zhou_2019.3	Captan	0.76	-0.68	[-2.24; 0.87]	2.3%
Zhou_2019.4	Captan	0.87	-1.12	[-2.73; 0.49]	2.2%
Mu_2013.2	Difenoconazole	1	0.43	[-1.64; 2.50]	1.8%
Zhou_2019.5	Captan	1	-0.68	[-2.23; 0.87]	2.3%
Lin_2021.1	Fluxapyroxad	1.1	-0.27	[-2.43; 1.89]	1.8%
Lin_2021.2	Fluxapyroxad	1.2	0.31	[-1.85; 2.48]	1.8%
Lin_2021.3	Fluxapyroxad	1.3	0.90	[-1.40; 3.20]	1.7%
Lin_2021.4	Fluxapyroxad	1.4	1.57	[-1.01; 4.15]	1.5%
Yang_2016b.1	Flutolanil	1.5	-1.45	[-3.12; 0.22]	2.2%
Mu_2013.3	Difenoconazole	1.5	1.37	[-0.80; 3.54]	1.8%
Lin_2021.5	Fluxapyroxad	1.5	1.05	[-1.30; 3.40]	1.6%
Lin_2021.6	Fluxapyroxad	1.6	1.32	[-1.14; 3.79]	1.5%
Qian_2018.1	Boscalid	1.7	0.13	[-1.09; 1.34]	2.6%
Yang_2016b.2	Flutolanil	1.8	-1.57	[-3.26; 0.13]	2.1%
Qian_2018.2	Boscalid	2	0.39	[-0.83; 1.61]	2.6%
Mu_2013.4	Difenoconazole	2	-0.52	[-2.59; 1.55]	1.8%
Yang_2016b.3	Flutolanil	2.16	-2.46	[-4.38; -0.53]	1.9%
Qian_2018.3	Boscalid	2.3	1.74	[0.41; 3.08]	2.5%
Teng_2019.2	Propiconazole	2.5	1.87	[-0.16; 3.91]	1.9%
Mu_2013.5	Difenoconazole	2.5	-1.74	[-3.98; 0.50]	1.7%
Yang_2016b.4	Flutolanil	2.59	-2.19	[-4.04; -0.34]	2.0%
Qian_2018.4	Boscalid	2.6	3.18	[1.61; 4.76]	2.2%
Qian_2018.5	Boscalid	2.9	3.26	[1.67; 4.86]	2.2%
Mu_2013.6	Difenoconazole	3	-2.35	[-4.73; 0.04]	1.6%
Yang_2016b.5	Flutolanil	3.10	-2.19	[-4.04; -0.34]	2.0%
Qian_2018.6	Boscalid	3.2	3.67	[1.99; 5.35]	2.2%
Teng_2019.3	Propiconazole	4.5	1.67	[-0.28; 3.63]	1.9%
**Random effects model**			**0.22**	**[-0.21; 0.66]**	**100.0%**
**Prediction interval**				**[-2.22; 2.67]**	

Heterogeneity: $I^2 = 73\%$, $\tau^2 = 1.43$, $p < 0.01$

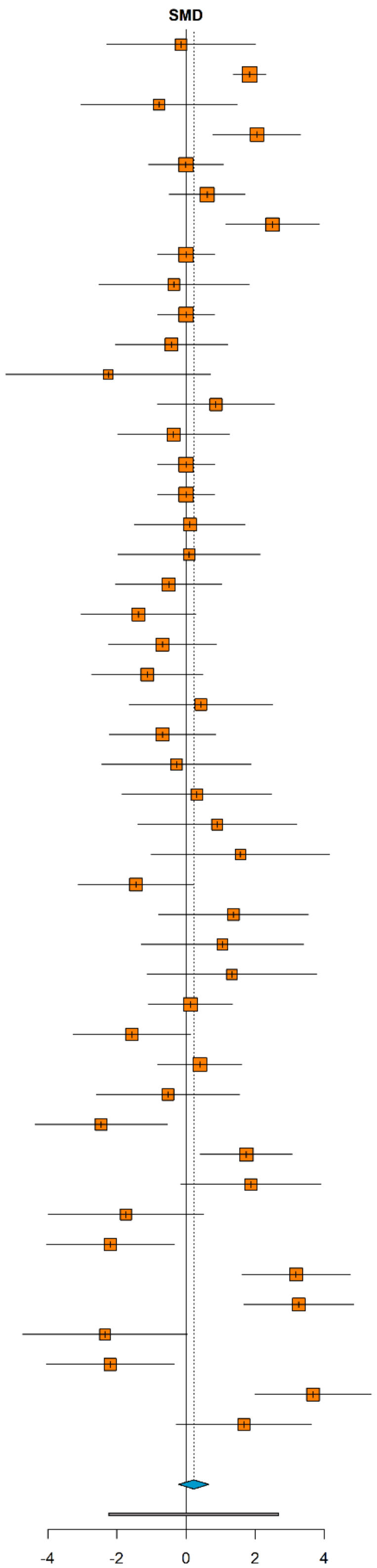

Fig. S10. The effect of exposure to fungicides on spontaneous movements in zebrafish excluding the studies by [65] and [68]. Data are presented as Hedges' G standardized mean differences (SMD) and 95% confidence intervals.
